# Flavonoids as Antidiabetic and Anti-Inflammatory Agents: A Review on Structural Activity Relationship-Based Studies and Meta-Analysis

**DOI:** 10.3390/ijms232012605

**Published:** 2022-10-20

**Authors:** Nur Farisya Shamsudin, Qamar Uddin Ahmed, Syed Mahmood, Syed Adnan Ali Shah, Murni Nazira Sarian, Muhammad Muzaffar Ali Khan Khattak, Alfi Khatib, Awis Sukarni Mohmad Sabere, Yusnaini Md Yusoff, Jalifah Latip

**Affiliations:** 1 Drug Discovery and Synthetic Chemistry Research Group, Department of Pharmaceutical Chemistry, Kulliyyah of Pharmacy, International Islamic University Malaysia, Indera Mahkota, Kuantan 25200, Pahang, Malaysia; 2 Department of Pharmaceutical Technology, Faculty of Pharmacy, Universiti Malaya, Kuala Lumpur 50603, Selangor, Malaysia; 3 Department of Pharmaceutical Engineering, Faculty of Engineering Technology (Chemical), Universiti Malaysia Pahang (UMP), Gambang Campus, Pekan 26300, Pahang, Malaysia; 4 Faculty of Pharmacy, Universiti Teknologi MARA Cawangan Selangor Kampus Puncak Alam, Bandar Puncak Alam 42300, Selangor, Malaysia; 5 Atta-Ur-Rahman Institute for Natural Product Discovery (AuRIns), Universiti Teknologi MARA Cawangan Selangor Kampus Puncak Alam, Puncak Alam 42300, Selangor, Malaysia; 6 Institute of Systems Biology (INBIOSIS), Universiti Kebangsaan Malaysia, Bandar Baru Bangi 43600, Selangor, Malaysia; 7 Department of Nutrition Sciences, Kulliyyah of Allied Health Sciences, International Islamic University Malaysia, Indera Mahkota, Kuantan 25200, Pahang, Malaysia; 8 Department of Biochemistry, Faculty of Biotechnology and Biomolecular Sciences, Universiti Putra Malaysia, Serdang 43400, Selangor, Malaysia; 9 Department of Chemical Sciences, Faculty of Science and Technology, Universiti Kebangsaan Malaysia, Bandar Baru Bangi 43600, Selangor, Malaysia

**Keywords:** flavonoids, antidiabetic, anti-inflammatory, structure–activity relationship study, correlation

## Abstract

Flavonoids are a group of naturally occurring polyphenolic secondary metabolites which have been reported to demonstrate a wide range of pharmacological properties, most importantly, antidiabetic and anti-inflammatory effects. The relationship between hyperglycaemia and inflammation and vascular complications in diabetes is now well established. Flavonoids possessing antidiabetic properties may alleviate inflammation by reducing hyperglycaemia through different mechanisms of action. It has been suggested that the flavonoids’ biochemical properties are structure-dependent; however, they are yet to be thoroughly grasped. Hence, the main aim of this review is to understand the antidiabetic and anti-inflammatory properties of various structurally diverse flavonoids and to identify key positions responsible for the effects, their correlation, and the effect of different substitutions on both antidiabetic and anti-inflammatory properties. The general requirement of flavonoids for exerting both anti-inflammatory and antidiabetic effects is found to be the presence of a C2–C3 double bond (C-ring) and hydroxyl groups at the C3’, C4’, C5, and C7 positions of both rings A and B of a flavonoid skeleton. Furthermore, it has been demonstrated that substitution at the C3 position of a C-ring decreases the anti-inflammatory action of flavonoids while enhancing their antidiabetic activity. Correlation is discussed at length to support flavonoids possessing essential pharmacophores to demonstrate equipotent effects. The consideration of these structural features may play an important role in synthesizing better flavonoid-based drugs possessing dual antidiabetic and anti-inflammatory effects. A meta-analysis further established the role of flavonoids as antidiabetic and anti-inflammatory agents.

## 1. Introduction

**Figure 1:**
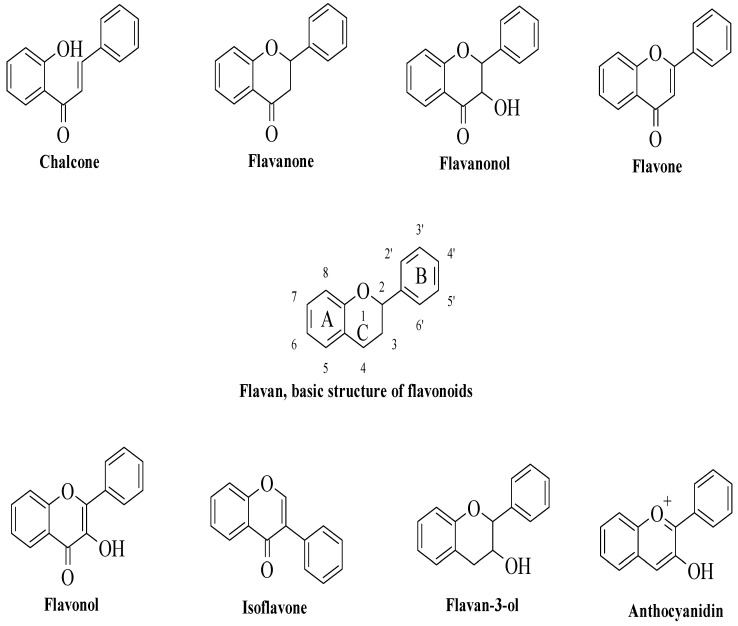
Flavonoids’ main classes.

Flavonoids are a type of plant secondary metabolite with a polyphenolic structure, and they are one of the most common families of natural products (NPs) [1,2]. Flavonoids exist naturally as aglycones, glycosides, and methylated derivatives, which are abundant in fruits, vegetables, and some beverages [1,2]. All flavonoids have fifteen carbon atoms in their fundamental nucleus C6–C3–C6 structure, with several chemical groups substituted. Flavonoids are categorized into chalcones, flavanones, flavanonols, flavones, flavanols, isoflavones, flavan-3-ols (catechins), and anthocyanidins based on their chemical structures, as illustrated in Figure 1. Flavonoids are a large family of NPs that have long been recognized as an essential component in a wide range of nutraceutical, pharmacological, medical, and cosmetic uses [1,2,3,4]. They are also vital substances with various health-promoting advantages for various disorders, including anticancer, antioxidant, anti-infective, antitoxic, hepatoprotective, anti-inflammatory, antidiabetic, and antiviral properties [3,4,5,6,7,8,9]. However, the anti-inflammatory and antidiabetic properties of flavonoids will be highlighted in this review, as the link between diabetes and inflammation has garnered interest among researchers [10,11]. 

Diabetes is one of the chronic diseases that can lead to death, and it was the seventh-highest cause of death in the United States in 2017 based on 83,564 death certificates [12]. According to WHO figures, diabetes was the direct cause of 1.5 million fatalities in 2019 [13]. Diabetes is a complicated metabolic illness that affects the body’s glucose levels. It arises when the pancreas produces insufficient insulin or when the body cannot efficiently use the insulin produced [10,13]. Hyperglycaemia, or elevated blood sugar, is a frequent complication of untreated diabetes and can cause catastrophic damage to various physiological systems, including the retina, kidneys, nerves, heart, and blood vessels [10,13]. Numerous concepts and hypotheses have been presented to elucidate the mechanisms typically involved in the pathophysiology of diabetes. One of the well-established views is that inflammation has a role in the development of diabetes [10,14]. In type 1 diabetes (T1D), the pancreas cannot produce enough insulin due to pancreatic beta-cell death. There are several inflammatory mediators involved during cell death, such as T-cell effectors directed against a variety of beta-cell autoantigens and related peptide epitopes; immune B cells undergo some modifications during illness progression; macrophages are essential mediators of islet inflammation by reactive oxygen species because of their direct toxicity to beta cells; dendritic cells, natural killer cells, and natural killer T cells may play a function in this pathophysiology process [10]. On the other hand, numerous cellular stressors that lead to inflammation are being hypothesised, which lead to insulin disorder and sensitivity in type 2 diabetes (T2D). The stressors include oxidative stress, reticular endoplasm stress, pancreas amyloid deposition, muscle, liver, and pancreatic ectopic deposition, gut microbiota, lipotoxicity, and glucotoxicity [10,15]. 

The rising significance of inflammation in T1D and T2D has increased interest in targeting inflammation to enhance disease prevention and treatment. Timely, encouraging preliminary results are shown in the clinical studies employing both T1D and T2D anti-inflammatory therapy, such as monoclonal antibodies and IL-1 antagonists, IKKbeta-NF-kappaB inhibitors (salsalate), and tumour necrosis factor (TNF) inhibitors, and supported the inflammation role in this context [10,15]. As previously discussed, flavonoids are one of the NPs that have been reported to exert both antidiabetic and anti-inflammatory effects. Based on several studies, flavonoids with antidiabetic characteristics can reduce inflammation via several pathways. The studies reported that apart from their antidiabetic effects, flavonoids such as quercetin, rutin, kaempferol, fisetin, morin, and luteolin also lower reactive oxygen species, proinflammatory signalling, oxidative stress [16,17,18], and lipotoxicity, which lead to improvement in inflammatory status [19,20,21,22,23,24]. It has been claimed that the biochemical features of flavonoids are structurally dependent but are not yet fully understood. As a result, the primary goal of this review is to identify critical locations responsible for the antidiabetic and anti-inflammatory activities of numerous structurally diverse flavonoids, their correlation, and the influence of alternative substitutions on the same properties. 

Distinguished scientific databases and search engines, namely, Google Scholar, Springer Link, Science Direct, Scopus, Wiley Online Library, PubMed, and Web of Science were thoroughly considered to find the relevant references and literature to complete this review. Additionally, the refereed non-indexed journals were also taken into consideration to collect all key information and to make certain that no well-documented information was left out. In this regard, a total of 102 articles from 2000 to 2022 focusing on diabetes, inflammation, polyphenolic compounds, flavonoids, the in vitro antidiabetic effects of flavonoids, the in vivo antidiabetic effects of flavonoids, the in vitro anti-inflammatory effects of flavonoids, the in vivo anti-inflammatory effects of flavonoids, the structure–activity relationship study of flavonoids for in vitro antidiabetic effects, the structure–activity relationship study of flavonoids for in vivo antidiabetic effects, the structure–activity relationship study of flavonoids for in vitro anti-inflammatory effects, and the structure–activity relationship study of flavonoids for in vivo anti-inflammatory effects, which were identified as keywords. After thorough study and investigations of all searched articles, 67 research manuscripts were finally selected to gather all the required information.

## 2. Anti-Inflammatory Activity of Flavonoids

**Figure 2:**
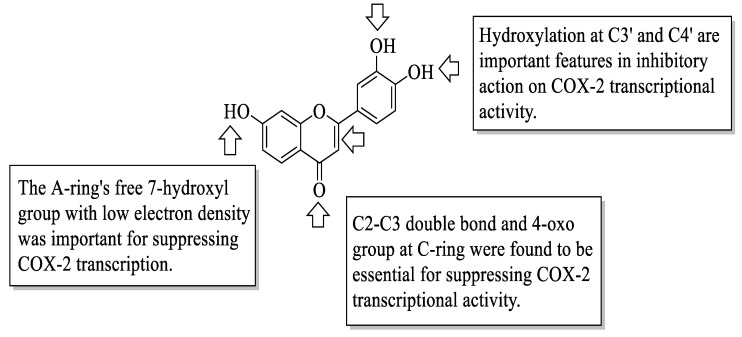
Structure of quercetin (**1**) and the labelled functional groups that are important in COX-2 inhibition [25].

Flavonoids have been reported to exert anti-inflammatory activity through different ways. Mutoh et al. evaluated the inhibitory activity of twelve different flavonoids on the transcription of the cyclooxygenase-2 (COX-2) gene in a human colon cancer cell line, namely DLD-1 cells [25]. The flavonoids included quercetin (**1**), rhamnetin (**2**), genistein (**3**), eriodyctiol (**4**), luteolin (**5**), kaempferol (**6**), fisetin (**7**), phloretin (**8**), catechin (**9**), epicatechin (**10**), epigallocatechin (**11**), and myricetin (**12**). It was discovered that **1** was the most potent COX-2 transcription suppressor, whereas **9** and **10** had the least inhibitory action. Briefly, **1** belongs to the flavonol class, and **10** belongs to the flavan-3-ol class; however, the presence of C2–C3 bond unsaturation and the oxygenation at C4 in **1** is thought to be responsible for its suppressing capabilities. It was also reported that the C2–C3 double bond of the C-ring, as can be seen in **4** and **5**, caused a minute effect on COX-2 transcriptional activity. Next, all tested flavonoids with a 4-oxo group were potent suppressors of COX-2 transcriptional activity, except **12**, which concluded that oxygen at C4 is critical for bioactivity. It was also corroborated by the fact that the compounds without oxygen at C4, such as **9**, **10**, and **11**, showed either minimum or no inhibitory effect. Subsequently, it was discovered that the number of hydroxyl groups on the B-ring may be substantial in COX-2 transcriptional activity. It was supported by the findings which stated that compounds containing hydroxyl groups at C3′ and C4′, such as **4**, **7**, **5**, **1**, and **2**, can significantly suppress COX-2 transcription. Meanwhile, compounds that bear three hydroxyl groups on the B-ring such as **11** and **12** had an absence of inhibitory activity. The free 7-hydroxyl group with low electron density in the A-ring was a significant structural feature for the suppression of COX-2 transcriptional activity. The authors concluded that the presence of a 4-oxo group in the C-ring, a 3′,4′-dihydroxy (catechol/1,2-dihydroxybenzene moiety) structure in the B-ring, and a low electron density in the 7-oxygen group in the A-ring are required for exerting anti-inflammatory activity via the suppression of COX-2 transcriptional activity, as shown in Figure 2. 

Next, flavonoids have also been reported to exert their anti-inflammatory activity through lipoxygenase (LOX) enzyme inhibition. Redrejo-Rodriguez et al. studied the inhibitory activity of LOX enzymes of four different flavonoids, namely, quercetin (**1**), luteolin (**5**), catechin (**9**), and taxifolin (**13**) [26]. It has been proposed that the flavonoids’ planar structure has a significant role in determining their inhibitory capacity. Additionally, it was also reported that the polar nature of flavonoid molecules did not prevent them from interacting with the LOX catalytic centre. Moreover, it was discovered that flavonoids’ LOX inhibitory effect is attributable to the C2–C3 double bond in the C-ring and the hydroxyl group at C-3′ and C-4′ in the B-ring. On the other hand, the presence of hydroxyl groups at C3 of the C-ring reduces the inhibitory activity.

**Figure 3:**
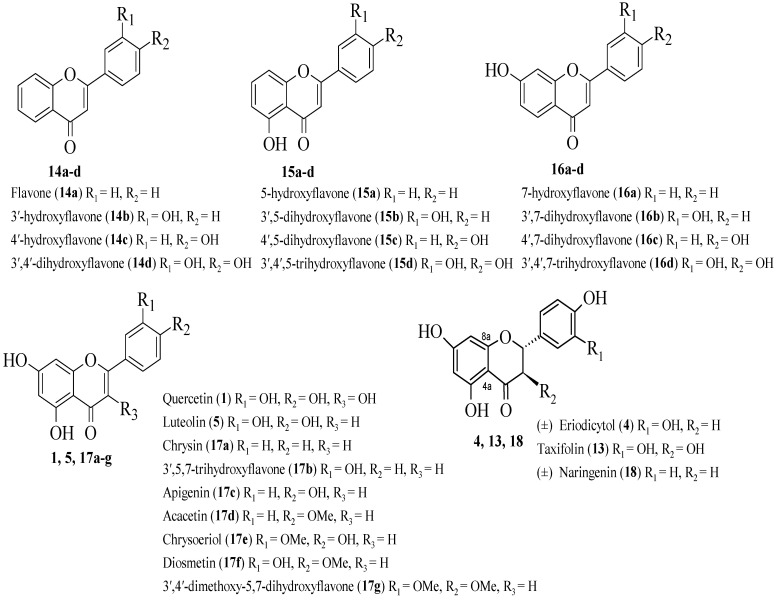
The chemical structures of studied flavonoids in inhibiting the formation of LTB4 [27].

Ribeiro et al. conducted a systemic investigation on the inhibition of the generation of leukotriene B4 (LTB4) and neutrophils by synthetic and natural flavonoids [27]. Figure 3 depicts the chemical structure of flavonoids that prevent the production of LTB4. In descending order of potency, the most active compounds were discovered to be luteolin (**5**), 3′,4′-dihydroxyflavone (**14d**), 3′,4′,7-trihydroxyflavone (**16d**), 3′,4′,5-trihydroxyflavone (**15d**), and quercetin (**1**). As reported by the authors, all the above-mentioned compounds contained catechol moiety in the B-ring, which may be responsible for the inhibitory activity. It was also reported that the number of hydroxyl groups in the A-ring did not appear to be a factor for the inhibition. However, the presence of a hydroxyl group at the C3 of the C-**17c** ring significantly reduced the LTB4 inhibitory activity, which was proved by compound **1** which exerted 2.5 times less potent inhibition than **5**. Next, **5** was shown to be more effective than **1**, possibly due to the latter’s high hydroxyl group count, which reduces hydrophobicity and inhibits flavonoid intercalation in the hydrophobic cavity that serves as the enzyme’s active site access channel. After that, the C2–C3 double bond appeared to be important for the inhibition as the tested flavanones had lower potency compared to flavones and flavonols. Cases **1** and **5** were reported to exhibit higher inhibition than non-planar compounds, **13**. Therefore, it can be said that planarity may influence flavonoids’ ability to interact with LOX enzymes. Notably, in order to demonstrate LTB4 enzyme inhibition, the structure–activity relationship study signalled the catechol group in the B-ring, the number and position of hydroxyl groups, the double bond between C2 and C3, and planarity appear to influence the inhibitory activity.

Additionally, using linoleic acid as a substrate, Sadik et al. studied the anti-inflammatory effects of 18 flavonoids on rabbit reticulocytes and soybean 15-LOX [28]. Firstly, it was discovered that the phenolic hydroxyl groups were not required for the inhibition. The hydroxyl group at C3 was found to be non-essential for inhibition, as evidenced by the luteolin’s (**5**) greater effectiveness than the quercetin (**1**). Apart from that, the presence of catechol moiety in the A-ring or B-ring can increase the inhibitory action. Following that, sugar moiety can significantly reduce the inhibition as it can decrease flavonoids’ hydrophobicity, which leads to a decrease in affinity towards LOX’s active site. The absence of the C2–C3 double bond of the C-ring appeared to decrease the inhibitory action as depicted by naringenin (**18**), hesperidin (**19**), epicatechin (**10**), and taxifolin (**13**). On the other hand, **1** that bears the C2–C3 double bond was reported to be more potent than the abovementioned flavonoids. The authors discussed that the C2–C3 double bond is significant as it completes a conjugated binding system that extends through all three rings and the carbonyl group of the C-ring, thereby stabilising the complexes or radical intermediates generated by flavonoids.

**Figure 4:**
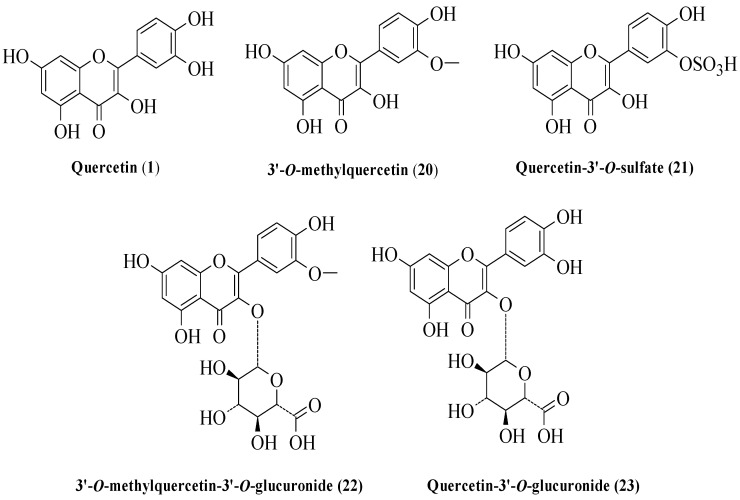
Quercetin and its metabolites inhibited inflammatory eicosanoid production from human leukocytes [29].

Similarly, Loke et al. compared the activity of quercetin (**1**) and its primary metabolites in suppressing inflammatory eicosanoid production from human leukocytes, demonstrating the role of metabolic transformation on flavonoid bioactivities [29]. Figure 4 shows **1** and its metabolites used in the test. Briefly, **1** potently inhibited LTB4 synthesis in leukocytes, and its activity was found to depend on the specific structural properties, specifically the C2–C3 double bond in the C-ring. It is a structural necessity for **1** to inhibit LTB4 since its absence eliminated the inhibitory activity. Furthermore, conjugation at the 3′-OH of **1** such as 3′-O-methylquercetin (**20**) and quercetin-3′-O-sulfate (**21**) decreased LTB4 inhibition by up to half, and glucuronidation at the 3-OH such as quercetin-3-O-glucuronide (**22**) and 3′-O-methylquercetin-3-O-glucuronide (**23**) also greatly diminished the LTB4 inhibitory action. Interestingly, the 3′-OH of the B-ring was more important in inhibiting LTB4 than the 3-OH of the C-ring. This was demonstrated when **1** was compared to structural analogues such as luteolin (**5**) and kaempferol (**6**) since the structural analogues exhibited a drop-in activity compared to **1** due to the absence of 3-OH in the C-ring (as seen in compound **5**) and 3′-OH in the B-ring (as shown in compound **6**).

**Figure 5:**
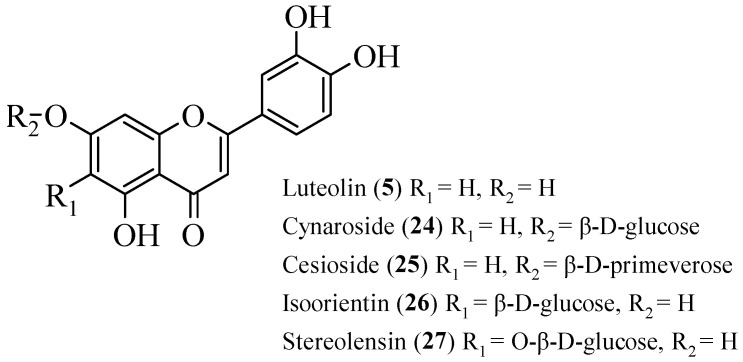
Flavonoids studied for their anti-inflammatory effect [30].

Odontuya et al. studied the SAR for the anti-inflammatory effect of flavonoids that were isolated from several plants [30]. There were five flavonoids that had been studied. Firstly, luteolin (**5**), cynaroside (**24**), and cesioside (**25**) were isolated from *H. corniculata.* Then, isoorientin (**26**) was obtained from *G. tenella* and *G. azurea,* as well as stereolensin (**27**) from *P. rotundifolia* and *P. incarnata*. The structures of these flavonoids are shown in Figure 5. All these phenolic compounds were tested against the synthesis of LTB4 and thromboxane B2 (TXB2). The results reported that **5** had the highest inhibition on both LTB4 and TXB2 synthesis compared to other compounds. Then, it was followed by **24** and **25**, with moderate inhibition on both syntheses. Meanwhile, **26** and **27** selectively showed good TXB2 inhibition. From the bioactivity studies, a structural activity relationship was summarized. For the non-selective inhibition of TXB2 and LTB4 synthesis, the authors discussed that in the A-ring, the presence of hydroxyl group at C7 and meta hydroxyls at C5 and C7 were important. In addition, the presence of ortho hydroxyl groups at C3′ and C4′ on B-ring also plays an important role in the inhibition. It was proved that the absence of these three characteristics will decrease the inhibition as demonstrated in **24** and **25**. For selective TXB2 synthesis inhibition, the structures of **26** and **27** were studied. It was reported that the direct attachment of the sugar moieties to OH- or carbon at C6 of the A-ring while retaining flavone basic hydroxyl groups will improve the inhibition against TXB2 synthesis.

Li et al. evaluated a *diverse* array of flavonoids 38 in number for their anti-inflammation potential through their COX-2 mRNA inhibition [31]. Then, a quantitative structure–activity relationship (QSAR) model was conducted to summarize the structural characteristics of flavonoids that were responsible for exerting good COX-2 mRNA inhibition. The results showed that the methoxy group at C4′ may increase the inhibition activity. In addition, the substitution of glucopyrasonyl at C8 instead of C6 can increase the COX-2 mRNA inhibition. It was proved that C-glycosylated luteolin (**5**) and apigenin (**17c**) showed greater inhibition compared to their isomers. In contrast, sugar substitutions and the -OH group at C3 caused lower COX-2 mRNA inhibition. Similarly, the C2–C3 double bond was also responsible for the decrease in inhibition.

**Figure 6:**
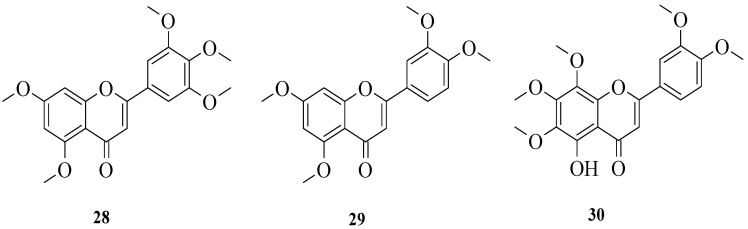
Chemical structures of flavonoids isolated from *M. paniculata* [32].

Wu et al. isolated three flavonoids from a famous traditional Chinese medicinal plant, *Murraya paniculata* (L.) Jack (Rutaceae) [32]. Then, the isolated phenolic compounds (Figure 6) were subjected to anti-inflammatory effect evaluation on the murine macrophage cell line and gastric epithelial cell (GES-1). The results reported that all compounds exerted anti-inflammatory effects. Based on the results, the authors summarized three structural characteristics that can improve anti-inflammatory effects. Firstly, methylation at the A-ring may influence the positive impact of the anti-inflammation of flavonoids. In contrast, the substitution of the methoxy group with other groups in the B-ring and the hydroxyl group in the A-ring will reduce anti-inflammatory activity. The influence of the hydroxyl group in the A-ring on reducing the activity can be seen in compound **30** as it had the weakest activity among the three compounds.

**Figure 7:**
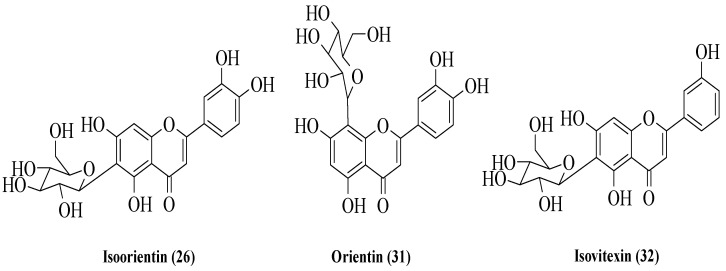
Chemical structures of flavonoids isolated from *V. grandifolia* [33].

Bello et al. examined three flavonoids isolated from *Vitex grandifolia* which is traditionally used by the Yoruba community in southwest Nigeria to treat various disorders [33]. The flavonoids were identified as isoorientin (**26**), orientin (**31**), and isovitexin (**32**) (Figure 7). The anti-inflammatory activity of the compounds was evaluated by using two different assays, viz., nuclear factor kappa B (NF-κB) inhibition and inducible nitric oxide synthase (iNOS) inhibition. The results showed that all compounds exerted good inhibition against NF-κB, with IC_50_ values of 8.9, 12, and 18 μg/mL for **26**, **31**, and **32**, respectively. On the other hand, only **32** exhibited moderate activity for iNOS inhibition with an IC_50_ of 21 μg/mL. Meanwhile, the other two compounds, viz., **26** and **31,** showed poor iNOS inhibition (IC_50_ = 48 and 54 μg/mL, respectively). The authors discussed the structure–activity relationship of these three flavonoids. Firstly, the C2–C3 double bond might influence the anti-inflammatory activity of flavonoids. Next, the -OH groups at C3′ and C4′, as shown in **26** and **31**, can increase the anti-inflammatory activity. In addition, the presence of sugar moiety in the A-ring compared to the B- and C-rings will lead to better anti-inflammatory activity.

López-Posadas et al. studied 14 flavonoids’ anti-inflammatory effects on rat splenocytes and carried out their structure–activity relationship [34]. Five different in vitro assays, viz., iNOS inhibition, COX-2 inhibition, the inhibition of cytokine secretion, antiproliferative activity on splenocytes, and the reduction in splenocytes’ viability were conducted to understand the anti-inflammatory effect of these flavonoids. Firstly, for iNOS inhibition, apigenin (**17c**), luteolin (**5**), and quercetin (**1**) portrayed complete inhibition; meanwhile, diosmetin (**17f**) and chrysin (**17a**) showed weak inhibition. On the other hand, hesperidin (**19**), kaempferol (**6**), genistein (**3**), and daidzein (**33**) were reported to be completely inactive against iNOS. For COX-2 inhibition, only **17c** and **17f** exerted inhibition. The authors discussed the structural requirements needed for both iNOS and COX-2 inhibition. It was highlighted that the presence of hydroxyl groups at the C2′ and C4′ of the B-ring may influence both activities. In contrast, methoxy and hydroxyl groups at C3 can reduce the iNOS inhibition. Secondly, for the inhibition of cytokine production such as TNF-α, IFN-γ, and IL-2, it was reported that all tested flavonoids showed good and effective inhibition. The authors summarized five structural requirements for the inhibition of cytokine production. The C2–C3 double bond, C4′ hydroxyl group, C3′ hydroxyl group, the absence of the C3 hydroxyl group, and the C5 hydroxyl group favoured the cytokine production inhibition. Moreover, the introduction of sugar moiety at C3 needs to be avoided as it causes a complete reduction in inhibition. Thirdly, similarly, all flavonoids showed excellent antiproliferative activity on both unstimulated splenocytes and concanavalin A-treated cells. It was also mentioned that about an average of 40% of splenocytes were reduced after the addition of the selected flavonoids. The C2–C3 double bond and the B-ring hydroxyl groups, particularly the C5 hydroxyl group, were reported to be important to these antiproliferative activities. Lastly, the reduction in splenocytes’ viability was also assessed. It was discussed that the **1**, **17c**, **5**, and **3** displayed stronger activity compared to other flavonoids. The authors mentioned that the C3′ and C4′ hydroxyl groups can increase the activity of the reduction in splenocytes’ viability.

Comalada et al. evaluated the effect of naturally occurring flavonoids viz. kaempferol (**6**), quercetin (**1**), apigenin (**17c**), chrysin (**17a**), diosmetin (**17f**), luteolin (**5**), daidzein (**33**), genistein (**3**), and hesperidin (**19**) on the macrophages derived from the bone marrow of mice [35]. The anti-inflammatory action on the macrophages and the structural activity analysis were conducted through three in vitro tests. Firstly, the antiproliferation effect on the macrophage colony-stimulating factor (M-CSF) was studied. The results showed that flavones (**17f** and **5**) and flavonols (**6** and **1**) exhibited the significant inhibition of M-CSF–macrophage proliferation. Meanwhile, isoflavones and flavanones displayed weak antiproliferative activity. Therefore, it can be concluded that the *iso*-position of the B-ring and the absence of the C2–C3 double bond can lead to lower antiproliferative activity. Secondly, the effect of flavonoids on the inhibition of TNF-α was conducted. It was reported that **5**, **1**, and **3** exerted good TNF-α inhibition. The authors discussed that the C3′ and C4′ hydroxyl groups as in luteolin and **1** favoured the inhibition. In addition, the one hydroxyl group as in **3** can also lead to better TNF-α inhibition. The author stressed that the absence of hydroxyl groups in the B-ring was detrimental and led to the negative results of TNF-α inhibition. Thirdly, iNOS inhibition showed that **1**, **17c**, **5**, and **17f** were able to inhibit iNOS and nitric oxide (NO) at low concentrations. The authors mentioned that the B-ring position, the C2–C3 double bond, and the hydroxyl group will influence the inhibition. Lastly, the authors suggested that **5** and **1** are the best naturally occurring flavonoids as anti-inflammatory agents due to the abovementioned characteristics.

Takano-Ishikawa et al. compared the structure–activity relationship of inhibition on lipopolysaccharide-induced prostaglandin E2 (PGE_2_) production in rat peritoneal macrophages between flavonoids subclasses [36]. There were 39 flavonoids studied, which were divided into five subclasses, namely flavones, flavonols, flavanones, isoflavones, and flavan-3-ols. It was reported that flavones, flavonols, flavanones, and isoflavones showed good inhibition of PGE_2_ production. In contrast, flavan-3-ol displayed weak inhibition. The C2–C3 double bond and 4-oxo of C-ring were discussed to be the important characteristics of a good inhibitor. In addition, the authors stressed that the absence of the C2–C3 double bond will lead to a loss of inhibition activities. When the tested flavonoids were compared, it was reported that the hydroxyl groups at the C5 and C7 positions led to a lower IC_50_ value, indicating better inhibition compared to the compounds without OH groups at C5 and C7. Similarly, in isoflavones alone, the hydroxyl group at C5 was reported to show better activity compared to compounds without the -OH residue at C5. Meanwhile, in flavones and flavonols, the absence of hydroxyl groups in the B-ring showed better inhibition compared to compounds that had OH groups such as at C3′ and C4′.

Kim et al. evaluated the effect of naturally occurring flavonoids on NO production in the macrophage cell line [37]. Among the 26 tested flavonoids, apigenin (**17c**) and luteolin (**5**) showed good inhibition with IC_50_ values of 23 and 27 μM, respectively. Meanwhile, naringenin (**18**) and apiin (**34**) showed the weakest inhibition with IC_50_ values up to 100 μM. The authors proposed that the C2–C3 double bond and the 5,7-dihydroxy groups at the A-ring favoured the inhibition of NO production. In addition, methoxy groups at C8 of the A-ring and the 4′ or 3′,4′-vicinal substitutions at the B-ring may also lead to better inhibition. In contrast, the 3-OH at the C-ring showed weaker activity which can be seen in flavonol derivatives such as flavonol, galangin (**35**), and quercetin (**1**). As mentioned before, **18**, a flavanone derivative, showed the weakest activity. Hence, it can be concluded that the C2–C3 double bond and the planar ring are important structural requirements to exhibit good inhibition of NO production.

**Figure 8:**
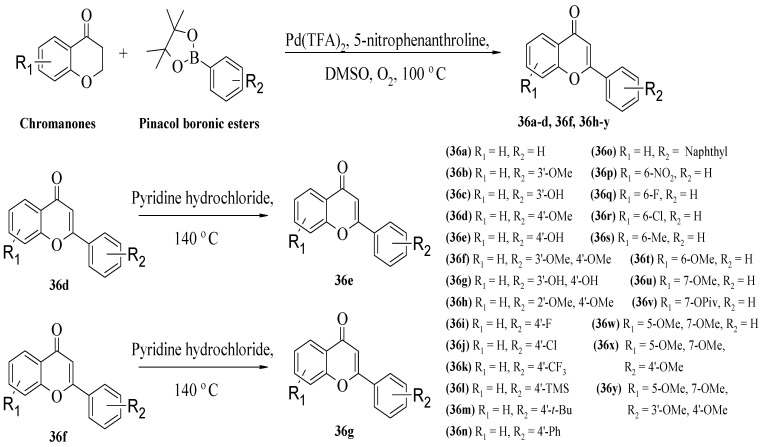
Synthesis pathway of new flavone derivatives and their chemical structures [38].

An et al. synthesized several flavone derivatives and evaluated their anti-inflammatory action via NO inhibition [38]. As depicted in Figure 8, the synthetic pathway was started with the reaction between chromanones and pinacol boronic esters. The final compounds were **36a–y**, and their chemical structures are demonstrated in Figure 8. It was reported that **36g** which contained hydroxyl groups at C3′ and C4′ of the B-ring showed maximum inhibitory action compared to other synthesized compounds. Therefore, the authors concluded that the catechol group may be responsible for NO inhibition. The statement was also supported by the fact that **36e** with a hydroxyl group at C4′ exerted better NO inhibition than **36f** with methoxy groups at C3′ and C4′. Halogenation at C4′ as shown in **36i**, **36j**, and **36k** either slightly lowered or showed no increase in the NO inhibitory action. Similarly, the authors reported that trimethylsilyl (TMS), alkyl, or aryl substituents at the C4′ position did not increase the inhibition action. Pivaloyloxy group at C7 of the A-ring and the hydroxyl groups at C5 and C7 as shown in **36v–y** exhibited moderate inhibitory action but still showed lower activity than **36g**, which possesses hydroxyl groups at the C3′ and C4′ positions. 

**Figure 9:**
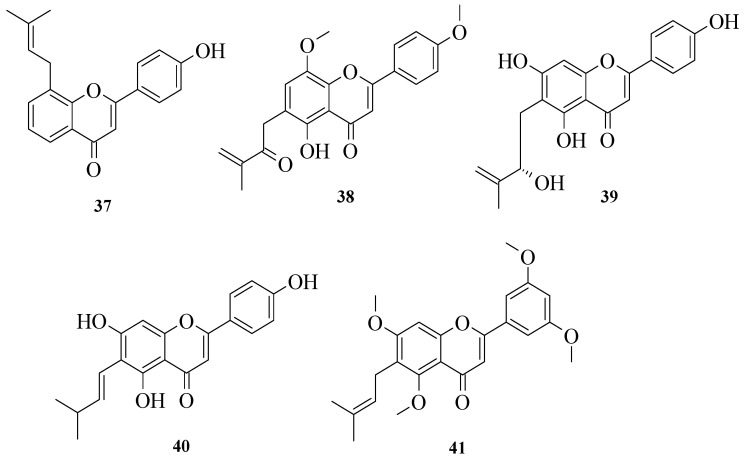
Chemical structure of isolated flavonoids from *A. heterophyllus* [39].

Liu et al. isolated several flavonoids from *Artocarpus heterophyllus* and tested their NO inhibitory activities [39]. The chemical structures of the isolated compounds are shown in Figure 9. The authors reported that compounds **39** and **40** exhibited significant inhibitory action compared to other isolated compounds. On the other hand, **38** and **41** were among the compounds that showed the weakest activities. Therefore, the authors concluded that the presence of hydroxyl groups at C5 and C7 of the A-ring, as well as at C4′ of the B-ring, were assumed to influence the flavonoids’ NO inhibitory activities.

Zhang et al. investigated the inhibition of IL-8 released by lipopolysaccharide-stimulated bronchial epithelial cells by 17 flavonoids [40]. It was found that apigenin (**17c**) and luteolin (**5**) exerted the most significant inhibition compared to other flavonoids. Both compounds contained hydroxyl groups at the C5 and C7 positions of the A-ring, the C2–C3 double bond, and oxygenation at C4 of C-ring. Therefore, it can be said that these structural features are important in the inhibitory activity. It was also discovered that chalcones with a broken C-ring, such as phloretin (**8**), exhibited equal efficacy to **17c**, indicating that the co-planar structure was not required or essential for inhibition. Pelargonidin (**42**), on the other hand, demonstrated poorer inhibition than **17c** and **8** in the absence of a C2–C3 double bond but with a closed C-ring, indicating that the C2–C3 double bond is a significant structural characteristic. Flavans, (+)-catechin (**9**), and (−)-epicatechin (**10**) with no C2–C3 double bond also had lower activity. After that, daidzein (**33**) and genistein (**3**) with the absence of hydroxyl groups at the C5 and C7 positions, respectively, had weak inhibition of LPS-induced IL-8 release. Therefore, the findings support the statement that the hydroxyl groups at the C5 and C7 positions of the A-ring are important for inhibitory action. Next, the authors mentioned that the hydroxyl group at the C3 position of the C-ring, as can be seen in naringenin (**18**), can improve inhibitory action. 

Ueda et al. evaluated the inhibition of flavonoids against TNF-α production and discussed the related structural features that are responsible for inhibition [41]. For an in vitro study, it was found that apigenin (**17c**), luteolin (**5**), quercetin (**1**), and myricetin (**12**) inhibited LPS-induced TNF-α that had been produced from a macrophage. Therefore, the authors hypothesized that the presence of hydroxyl groups at the C5 and C7 positions of the A-ring, as well as at the C4′ position of the B-ring, seemed to be important features. Moreover, galangin (**35**) and chrysin (**17a**) exhibited weaker and an absence of inhibitory activities, respectively, when compared. Therefore, it can be suggested that an absence of the hydroxyl group at C4′ of the B-ring can reverse or reduce the bioactivity. Following that, for in vivo assay, the tested flavonoids were orally supplied to mice. It was reported that **5** and **17c** decreased serum TNF-α production after oral administration. The presence of hydroxyl groups at the C4′ (B-ring), C5, and C7 (A-ring) positions seemed to be important for oral inhibition, as both **5** and **17c** contained these features. Furthermore, as **5** exhibits stronger inhibition, the hydroxyl group at the C3′ position of the B-ring may be responsible for the greater inhibition observed with **5**. 

**Figure 10:**
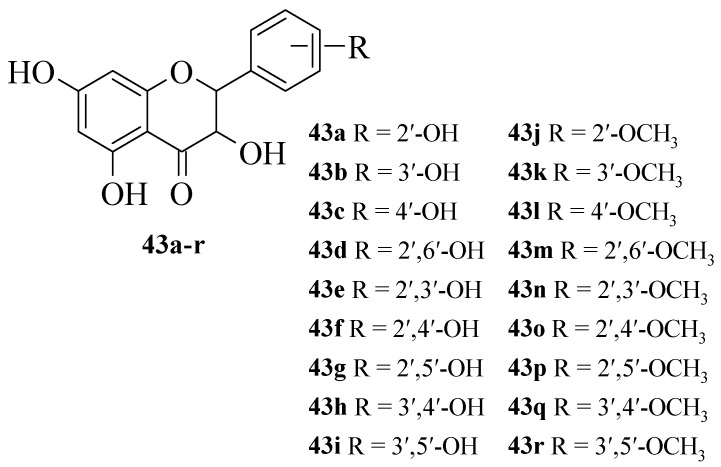
Chemical structures of new flavanonol derivatives [42].

Jiang et al. synthesized 19 flavanonols and evaluated their NO inhibitory action in RAW macrophage 264.7 cells, as well as discussed the structural activity relationship [42]. The synthesized compounds are shown in Figure 10. It was reported that **17e** with dihydroxy groups at C2′ and C3′ exerted the maximum NO inhibition when compared to other synthesized compounds. Briefly, **43a**, **43b**, and **43g** that contained one hydroxyl group either at C2′ or C3′ also exhibited significant inhibition, albeit weaker than **43e**. Therefore, it was proved that hydroxyl groups at C2′ and C3′ are important structural requirements for NO inhibition. The fact was further corroborated when the methylation of C2′-OH, C3′-OH, or both C2′ and C3′-OH, as can be seen in **43j**, **43k**, and **43n**, respectively, caused inactivity. It was also reported that substitution at the C4′ position, as seen in **43c**, **43f**, and **43h**, can lead to weaker inhibitory action. 

**Figure 11:**
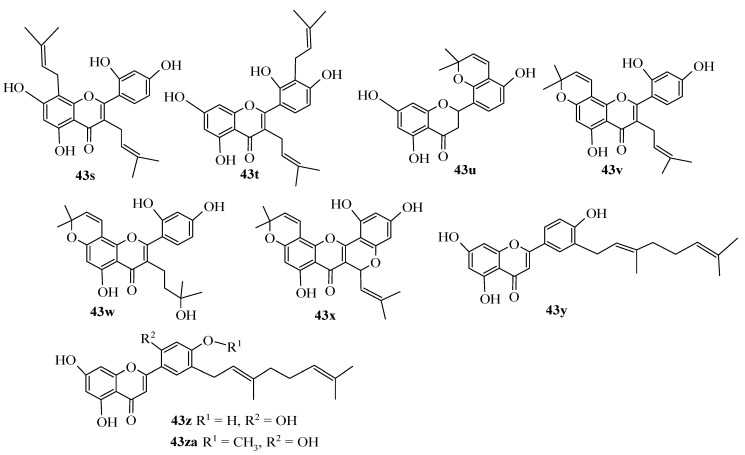
Chemical structures of flavonoids isolated from the root bark of *Morus alba* L. [43].

Culenova et al. isolated several prenylated flavonoids from the root bark of *Morus alba* L. (Moraceae), commonly known as mulberry. The isolated prenylated flavonoids, namely, kuwanon C (**43s**), kuwanon T (**43t**), sanggenon H (**43u**), morusin (**43v**), morusinol (**43w**), cyclomorusin (**43x**), kuwanon S (**43y**), kuwanon E (**43z**), and kuwanon U (**43za**) (Figure 11) were evaluated for their in vitro anti-inflammatory ability to inhibit cyclooxygenase 2 (COX-2). Of the prenylated flavonoids tested in this study, kuwanon E showed the most potent inhibitory effect. The replacement of the hydroxyl group at C-4′ by a methoxyl in the compound kuwanon U diminished the in vitro inhibitory activity against COX-2, confirming the importance of a polar functional group at this position. Kuwanon E showed significantly better inhibitory activity than kuwanon C (*p* < 0.01), morusinol (*p* < 0.01), and cyclomorusin (*p* < 0.05). It was revealed that flavonoids with an isoprenyl group at C-3 (including **43s**) were weak COX-2 inhibitors with less activity than the reference inhibitor, i.e., indomethacin. The results also showed that, in comparison to **43s**, modifications of the isoprenyl moieties at C-3 and C-8 in **43w** did not significantly influence the activity [43].

Lin et al. further evaluated the anti-inflammatory and antioxidative activities of mulberry (*M. alba* L.) leaf flavonoids. Initially, different ethanol concentration (30%, 50%, and 75%)-based extracts were prepared to obtain flavonoid-rich extracts. These extracts inhibited the production of nitric oxide (NO), prostaglandin E2 (PGE_2_), inducible nitric oxide synthase (iNOS), cyclooxygenase-2 (COX-2), and inflammatory cytokines in lipopolysaccharide (LPS)-induced RAW 264.7 cells. All extracts increased the antioxidative capacity by decreasing the reactive oxygen species (ROS) production and the scavenging of 2,2-diphenyl-1-picrylhydrazyl (DPPH) free radicals and improving the metal ion chelating activity and reducing power. The results revealed that the extract prepared using 30% ethanol exhibited the best anti-inflammatory and antioxidative activities. The LC-MS analysis revealed the presence of 24 different flavonoids in the resultant extracts. Finally, a nontargeted metabolomic analysis confirmed that quercetin (**1**), kaempferol (**6**), and their derivatives in 30% ethanol extract were more abundant than the other two extracts and may be the main flavonoids involved in anti-inflammatory and antioxidative effects. Furthermore, 30% ethanol extract of mulberry leaf was also evaluated for the pharmacological activities in dextran sodium sulfate (DSS)-induced ulcerative colitis (UC) mice. The same extract alleviated the clinical symptoms, reduced the secretion of inflammatory cytokines, and inhibited the activation of the inflammatory pathway in DSS-induced colitis mice [44]. 

Another interesting structure–activity relationship-based study was conducted by Wang et al. to uncover the anti-inflammatory potential of flavones. They investigated and summarized commonly applied in vitro, in vivo, and clinical models in testing the anti-inflammatory activity of flavones. They systematically mapped the anti-inflammatory structure–activity relationship of flavones and performed the cross-comparisons of that with flavanones, flavanols, and isoflavones. They found out that the hydroxyl groups (-OH) are indispensable for the anti-inflammatory function of flavones, and -OH at the C-5 and C-4′ positions were found to enhance, while -OH at the C-6, C-7, C-8, and C-3′ positions was found to diminish their activity. Moreover, the C2–C3 single bond and -OH at the C-3 and B-ring positions weakened flavone aglycones’ activity. It was also discovered that most of the flavone aglycones showed activity through the NF-κB, MAPK, and JNK-STAT pathways, and their possible cell binding targets were found to be kinase, aryl hydrocarbon receptor (AhR), G-protein coupled receptors, and estrogen receptors [45].

## 3. Antidiabetic Activity of Flavonoids

**Figure 12:**
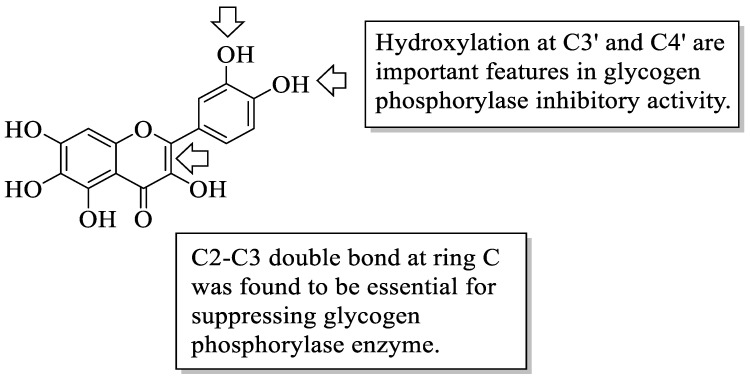
The structure of **44** and the functional groups responsible for the inhibition of glycogen phosphorylase [46].

Flavonoids have been reported to demonstrate antidiabetic effects through various molecular mechanisms of action. Kato et al. investigated the structural requirements of flavonoids with glycogen phosphorylase inhibitory activity. Glycogen phosphorylase is one of the enzymes that catalyse the breakdown of glycogen into glucose in the liver, and its inhibition has been shown to modulate the glucose level associated with T2D [46]. The researchers discovered a flavone called quercetagetin (3,3′,4′,5,6,7-hexahydroxyflavone (**44**), which bears hydroxyl groups at six different positions to be the most efficient of all the flavonoid compounds examined. The positions include C3 of the C-ring, C3′ and C4′ of the B-ring as well as C5, C6, and C7 of the A-ring. After that, it was summarized that the presence of hydroxyl groups at C3′ and C4′ in the B-ring and the C2–C3 double bond were found to be critical variables for inhibition, as is illustrated in Figure 12.

Matsuda et al. studied the aldose reductase inhibitory action of flavonoids and the structure–activity relationship [47]. Aldose reductase is a central enzyme in the polyol pathway that has been shown to catalyze glucose reduction to sorbitol, which has been linked to a diabetic impact. There are several important structural features that have been discussed by the authors. Firstly, it was found that flavones with no hydroxyl group at position C5 of the A-ring had equivalent bioactivity when compared to chrysin (**17a**) that is bearing C5-OH. It was hypothesized that the hydroxyl group at C5 may not be essential for inhibition against aldose reductase. Then, it was reported that diosmetin 7-O-β-D-glucopyranoside (**45**) had weaker inhibition compared to diosmetin (**17f**). Therefore, it can be assumed that 7-O-glucosyl moiety can lead to a reduction in inhibitory action. The presence of the hydroxyl group at C3 was shown to reduce inhibition, which is supported by the fact that 3-O-methyl or 3-O-monosaccharide derivatives are better inhibitors than the comparable free flavonols at the C3 position. Next, apigenin (**17c**) inhibited more effectively than kaempferol (**6**), implying that the presence of the 3-OH group is not essential for the higher inhibition. Lastly, the authors mentioned that flavonoids with catechol moiety at the B-ring (hydroxyl groups at C3′ and C4′) demonstrated better inhibition than flavonoids with pyrogallol moiety (hydroxyl groups at C3′, C4′, and C5′ positions).

Matsuda et al. [48] further investigated the structural requirements of flavonoids for the suppression of advanced glycation end-products (AGEs) production. AGEs are one of the consequences of persistent hyperglycaemia, a condition that diabetic individuals endure. It was proposed that increasing the number of hydroxyl groups at C3′ and C4′ of the B-ring, as well as the C5 and C7 locations of the A-ring, can increase flavones’ inhibition against AGE production. Following that, the methylation or glycosylation (i.e., the introduction of sugar moiety) of the hydroxyl group at C3′ or C4′ can reduce the inhibitory action of AGE production. It was also shown that the direct attachment of a sugar moiety to the OH group of the C7 position at the A-ring of flavones and isoflavones decreased inhibitory action. However, the methylation of the flavonols hydroxyl group at C3 of the C-ring appeared to boost activity.

Next, Matsuda et al. investigated the effect of 44 flavonoids on the adipogenesis of 3T3-L1 adipocyte cells [49]. The structural analysis that had been summarized by the authors reported that most flavonoids bearing hydroxyl groups lacked the effect of promoting the accumulation of triglyceride (TG), which acts as a marker of adipogenesis. However, flavonols with methoxy groups exerted a stronger escalation of TG concentration, especially those with a methoxy group at the C3 position. Flavonol’s methoxy group at the B-ring was also found essential for increasing TG.

**Figure 13:**
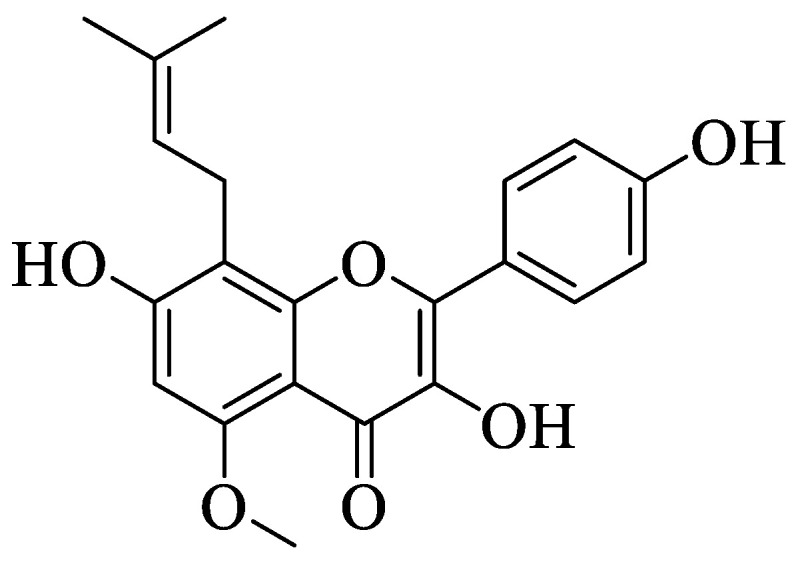
Chemical structure of **46** [50].

Jung et al. investigated a prenylated flavonol called sophoflavescenol (**46**) (Figure 13) for its antidiabetic potential [50]. Briefly, **46** was extracted from a Northeast Asian perennial shrub called *Sophora flavescens* Ait. This study experimented with the inhibition of **46** against rat lens aldose reductase (RLAR), human recombinant aldose reductase (HRAR), and advanced glycation end products (AGE). For RLAR inhibition, **46** showed a significant IC_50_ value (0.30 μM) when compared to the control, epalrestat (0.07 μM). For HRAR inhibition, **46** also showed a remarkable IC_50_ value (0.17 μM) compared to the control, epalrestat (IC_50_ 0.15 μM). Meanwhile, for AGE inhibition, **46** portrayed a stronger inhibition with a lower IC_50_ value (17.89 μg/mL) when compared to the control, aminoguanine (IC_50_ 81.05 μg/mL). The authors discussed that there are three important structural characteristics that contributed to the remarkable RLAR, HRAR, and AGE inhibitions. Firstly, flavonols with a prenyl group at the C8 position and C3′,4′-dihydroxyl groups lead to more potent inhibition. Next, the presence of the methoxy group at C-5 also caused stronger inhibition. Lastly, the essential structural characteristic that contributed to the strong inhibition is the presence of the hydroxyl group at the C-4′ position.

**Figure 14:**
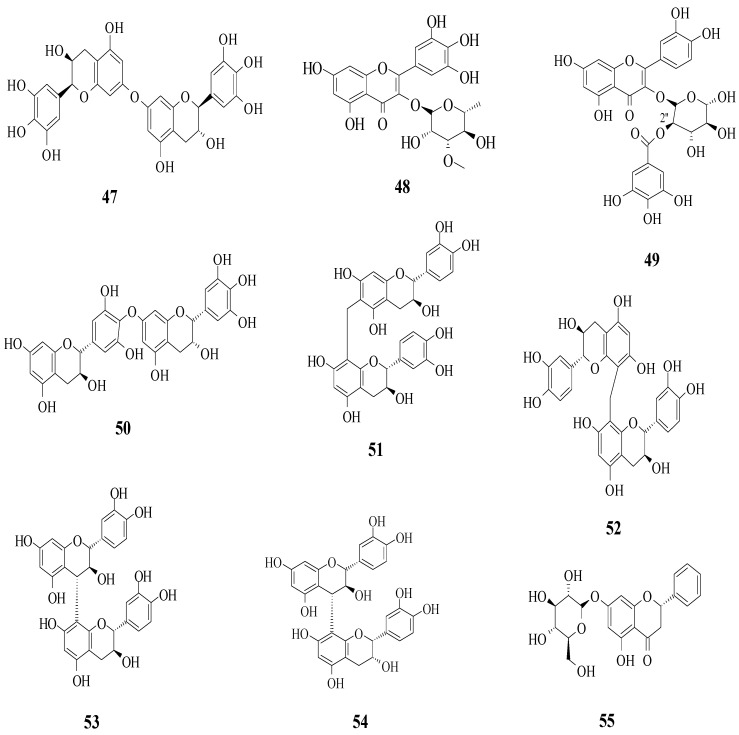
The flavonoids isolated as α-glucosidase inhibitors from *P. anserine* [51].

Yang et al. reported the identification of 30 different phenolic compounds from the rhizomes of *Potentilla anserina* L. [51]. All compounds were tested for their α-glucosidase inhibitory effect by using acarbose as a positive control. As it is well known, inhibiting α-glucosidase is critical in the management of T2D because α-glucosidase catalyses the hydrolysis of starch to simple sugar. All compounds that have been discussed here are displayed in Figure 14. It was reported that several prominent structural characteristics play an important role in stronger inhibition. Firstly, the dimerization of flavonoids was found to be responsible for stronger inhibition. It was proved that compounds **47** and **50**–**54** belong to the biflavonoid category and portrayed remarkable IC_50_ values, which ranged from 2.57 to 8.96 μM. Next, the substitution of the gallolyl moiety instead of the glucose moiety at C-2″ can significantly improve the inhibition activity. It can be seen in compound **49** with the gallolyl moiety at C-2″ (IC_50_ = 1.05 μM) compared to compounds **48** with the glucose moiety and **1** (82.47 and 75.80 μM). The presence of a hydroxyl group in the B-ring was discovered to be essential, as evidenced by compound **55′**s weak inhibition (IC_50_ = 155.57 μM).

**Figure 15:**
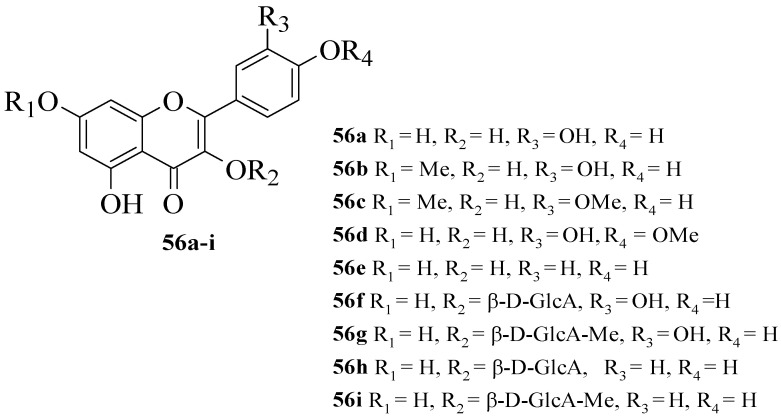
The structures of flavonoids isolated from *T. gallica* [52].

Hmidene et al. studied the effect of five simple flavonols **56a**–**e** and glucuronirated flavonols **56f**–**i** from *Tamarix gallica* on α-glucosidase inhibitory activity [52]. The acarbose was used as a positive control. The chemical structures of all the compounds tested are elucidated in Figure 15. It was reported that all nine compounds showed a dose-dependent inhibition and portrayed higher inhibition compared to acarbose. Based on compounds **56a** and **56e**, it was suggested that the hydroxyl group at C3′ with glucuronic acid and methyl ester was responsible for α-glucosidase inhibition.

**Figure 16:**
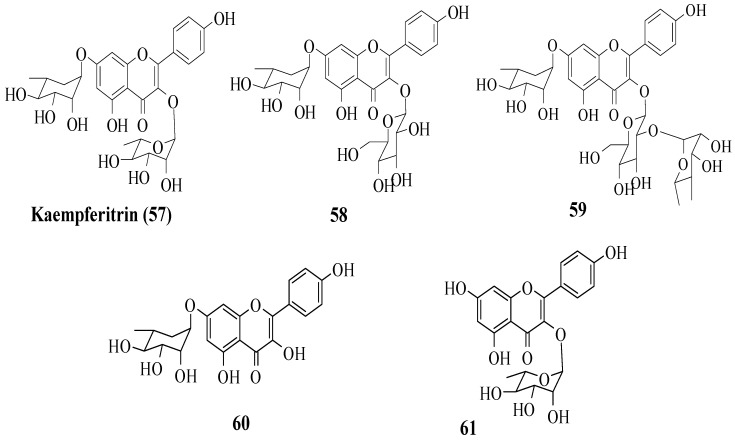
The structures of flavonoids isolated from *S. dendroideum* [53].

Da Silva et al. investigated the in vivo antidiabetic activity of kaempferol derivatives that were isolated from *Sedum dendroideum* leaf extract [53]. There were five derivatives tested in streptozotocin-induced diabetic mice for acute hypoglycaemic activity. The compounds tested are shown in Figure 16. It was reported that rhamnosyl units at positions 3 and 7 were responsible for the hypoglycaemic activity. In other words, a rhamnosyl unit at position C3 is important as it is present in kaempferitrin (**57**) but not in **58** and **59**, and the results showed **57** had higher hypoglycaemic activity compared to **58** and **50**. Next, to test the importance of the rhamnosyl unit at C7, **60** (with rhamnosyl unit at C7) and **61** (without rhamnosyl unit at C7) were tested. The results supported that a rhamnosyl unit at C7 is important as **60** exhibited hypoglycaemic activity after 120 min, but **61** showed activity after 60 min and lost activity at 120 min.

**Figure 17:**
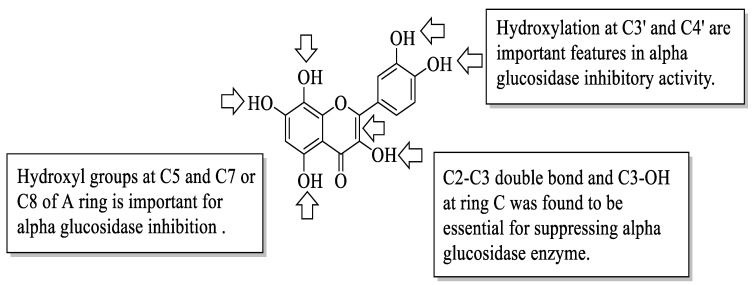
The SAR of flavonoids for α-glucosidase inhibition activity [54].

Proença et al. evaluated the series of 40 flavonoids for an in vitro α-glucosidase inhibition. The compounds were grouped into five groups [54]. After testing all the compounds’ α-glucosidase inhibitory activity against acarbose, the authors created a pattern of structural characteristics that were responsible for the activity. The pattern was created based on the most active compounds with IC_50_ values of 7.6 ± 0.4 μM and 15 ± 3 μM. Based on Figure 17, the presence of hydroxyl groups at the C5 and C7 or C8 positions of the A-ring is important. Next, the hydroxyl groups at C3′ and C4′ of the B-ring were also important for the inhibition. Then, in the C-ring, the C2–C3 double bond and the hydroxyl group at C3 were important. Furthermore, the authors mentioned that the position and amount of hydroxyl groups were the determinants for the α-glucosidase inhibition of flavonoids.

**Figure 18:**
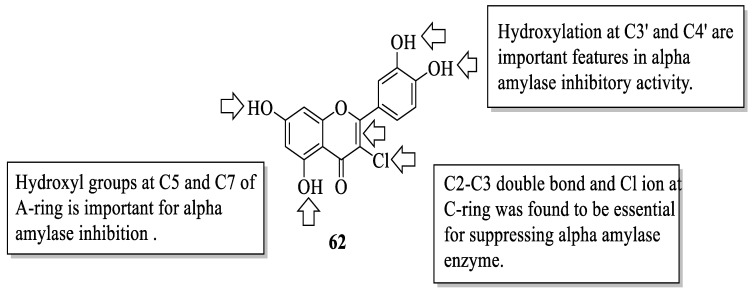
The SAR of flavonoids for α-amylase inhibition activity [55].

Similarly, Proença et al. studied the same series of 40 flavonoids. However, the bioactivity tested was slightly different, in which inhibition against pancreatic α-amylase was evaluated [55]. Like α-glucosidase inhibition, α-amylase inhibition is also considered one of the T2D management, as it catalyses the hydrolysis of starch to simple sugar. Acarbose was also used in this study as a positive control. It was revealed that the compound with the most effective inhibition was **62** (3-chloro-3′,4′,5,7-tetrahydroxyflavone) with an IC_50_ value of 44 ± 3 μM. Then, based on this most-active compound, an activity pattern was created by the authors. Based on Figure 18, it was found that the presence of a Cl atom at C3 and the C2–C3 double bond of the C-ring was important for strong inhibition. Furthermore, the presence of hydroxyl groups at C5 and C7 of the A-ring, as well as C3′ and C4′ of the B-ring was also responsible for α-amylase inhibition. In addition, the authors discussed that the position and nature of substituents were also the determinants for the α-amylase inhibition of flavonoids.

**Figure 19:**
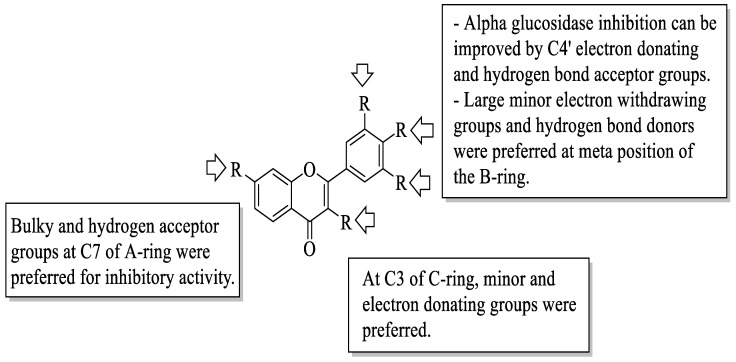
The SAR of flavonoids for α-glucosidase inhibition activity [56].

Next, Jia et al. studied several dietary flavonoids for their α-glucosidase inhibitory and insulin-sensitizing potentials [56]. For α-glucosidase inhibition, 27 dietary flavonoids were tested against a positive control, acarbose. The results revealed that three compounds demonstrated remarkable inhibition based on the IC_50_ value. The values reported were myricetin (**12**) (IC_50_ = 11.63 ± 0.36 μM) > apigenin-7-O-glucoside (**63**) (IC_50_ = 22.80 ± 0.24 μM) > fisetin (**7**) (IC_50_ = 46.39 ± 0.34 μM). Then, by using the 3D-quantitative structure-activity relationship model, structural characteristics that were needed for good inhibition were summarized. There are four important characteristics that can be seen in Figure 19. Firstly, an electron-donating group and hydrogen bond acceptor groups at C4′ of the B-ring can improve the inhibition. In the same B-ring, bulky, minor, electron-withdrawing groups and hydrogen bond donors were favoured at the *meta*-position. Next, minor and electron-donating groups, as well as hydrogen bond donor groups, were favoured at C3 of the C-ring. After that, at C7 of the A-ring, bulky and hydrogen acceptor groups were favoured. Then, for insulin sensitization activity, all compounds were tested by using molecular docking and in vitro evaluation with insulin-resistant HepG2 cells. The results showed five flavonoids that exerted good insulin sensitization activity which were baicalein (**64**), isorhamnetin-3-O-rutinoside (**65**), **63**, kaempferol-7-O-β-glucoside (**66**), and cyanidin-3-O-glucoside (**67**). There was no structural analysis conducted on these five flavonoids for insulin sensitization. However, from both studies, the authors concluded that compound **63** can be used in diabetes management in the future as it exerted excellent activity in both α-glucosidase inhibition and insulin sensitization.

**Figure 20:**
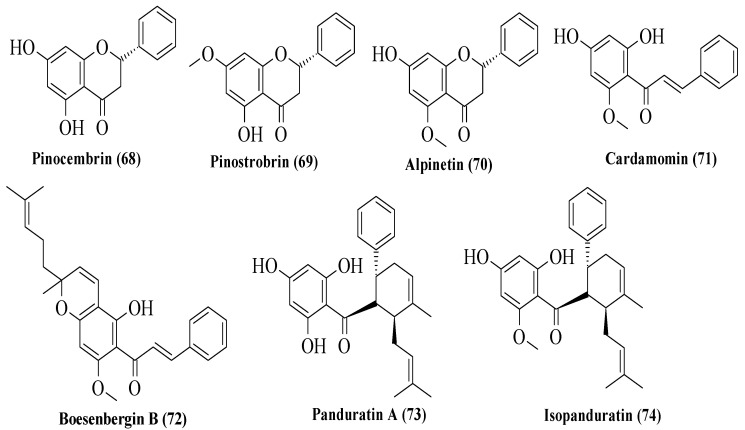
The structures of flavonoids isolated from *B. rotunda* [57].

Potipiranun et al. isolated several flavonoids to test their activities on antidiabetic complication (AGE inhibition) and α-glucosidase inhibition [57]. Three flavanones, two chalcones, and two dihydrochalcones were isolated from *Boesenbergia rotunda*, which is known as fingerroot. Based on Figure 20, flavanones consist of pinocembrin (**68**), pinostrobin (**69**), and alpinetin (**70**). Meanwhile, chalcones are cardamomin (**71**) and boesenbergin B (**72**), whereas dihydrochalcones are panduratin A (**73**) and isopanduratin (**74**). For the evaluation of AGE inhibition, two methods were conducted, namely AGE inhibition assay and methylglyoxal (MG) trapping activity. MG is a known precursor of glycation. It was reported that most compounds showed greater AGE inhibition than the control, aminoguanidine. Then, for MG trapping activity, all compounds showed comparable activities compared to aminoguanidine. Briefly, **68** was the most active compound for MG trapping activity, with an EC_50_ value of 63.22 ± 10.12 µM. By using the structure of **68** and other compounds, the SAR of flavonoids on the MG trapping activity was summarized. Firstly, hydroxy groups can improve the inhibition, while methoxy and geranyl groups can reduce the inhibition. Next, the presence of a methoxy group at the C7 position of dihydrochalcone can improve the activity compared to methoxy groups at the C5 position. After that, for α-glucosidase inhibition, only bioactivity studies were conducted, while no SAR was conducted. It was reported that **68** also demonstrated an inhibitory effect against α-glucosidase.

**Figure 21:**
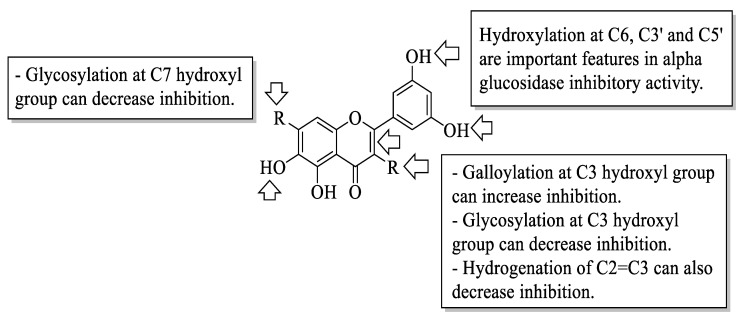
The SAR of flavonoids for α-glucosidase inhibition activity [58].

Xiao et al. reviewed an α-glucosidase inhibitory effect of dietary flavonoids and summarized important structural characteristics that were responsible for the inhibition [58]. The summary of the characteristics that influenced the inhibition is illustrated in Figure 21. Based on Figure 21, the presence of hydroxyl groups at C6 of the A-ring and C3′ and C5′ of the B-ring can increase the activity of flavonoids on α-glucosidase inhibition. Next, the galloylation of the hydroxyl group of C3 of the C-ring can also influence the inhibition. In contrast, the hydrogenation of C2=C3 of the C-ring, as well as the attachment of sugar moiety to the hydroxyl groups of C3 of the C-ring and C7 of the A-ring, would decrease the inhibitory activity.

**Figure 22:**
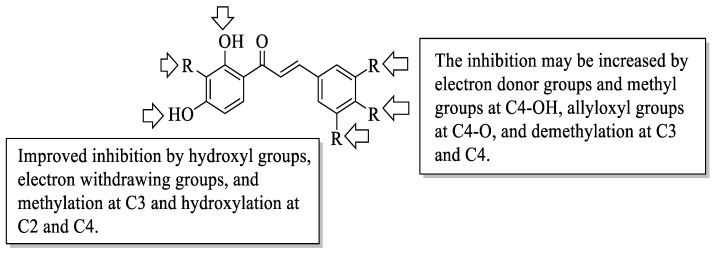
SARs of chalcones for PTP1B inhibition activity [59].

**Figure 23:**
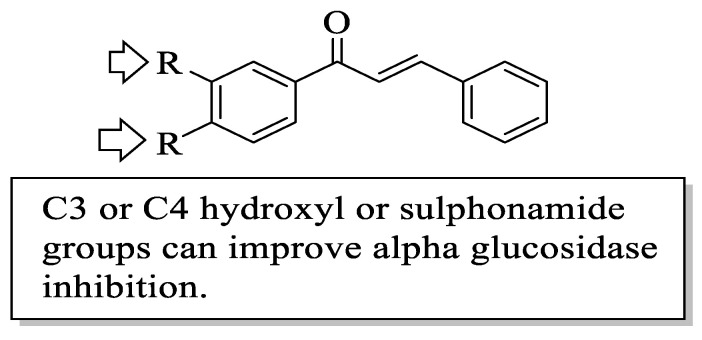
SARs of chalcones for α-glucosidase inhibition activity [59].

**Figure 24:**
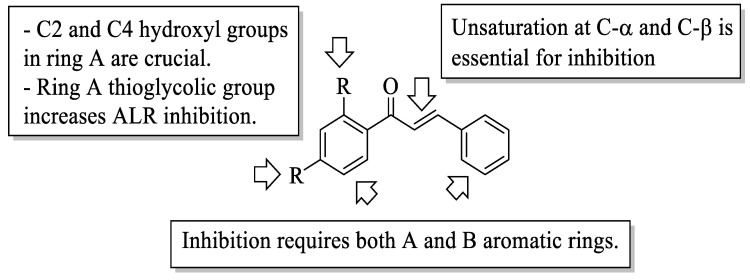
SARs of chalcones for ALR inhibition activity [59].

**Figure 25:**
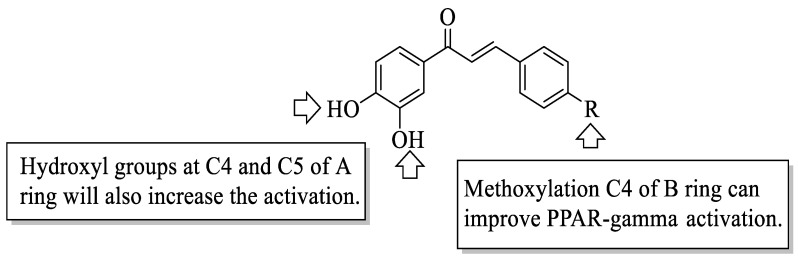
SARs of chalcones for PPAR-gamma activation activity [59].

Mahapatra et al. reviewed all SAR studies that were related to the antidiabetic activities of chalcones from 1977 to 2014 [59]. Then, the authors narrowed down the activities into four different effects, namely, the protein tyrosine phosphatase 1B (PTP1B) inhibitory effect, the α-glucosidase inhibitory effect, the aldose reductase (ALR) inhibitory effect, and the peroxisome proliferator-activated receptor (PPAR) gamma-activating effect. The role of α-glucosidase and ALR was discussed previously. Meanwhile, PTP1B is a prime enzyme responsible for insulin receptor desensitization, and the activation of PPAR gamma plays a critical role in glucose homeostasis by regulating cellular differentiation and development, and the metabolism of carbs, lipids, and proteins [60,61]. All structural characteristics that influenced the bioactivities have been summarized and illustrated. Firstly, based on Figure 22, there are several characteristics that influenced the PTP1B inhibition of chalcones. The hydroxyl groups, electron-withdrawing groups, methylation at C3′, and the hydroxyl groups at C2′ and C4′ of the A-ring can improve the inhibition. Meanwhile, electron-donating groups; methyl groups substitution with the -OH of C4; allyloxyl groups at the -O of C4; demethylation at C3 and C4; and allyl group at C5 of the B-ring may increase the inhibition. After that, for α-glucosidase inhibition, based on Figure 23, hydroxyl groups and sulphonamide groups at C3′ or C4′ of the A-ring can lead to better inhibition. Next, for ALR inhibition, four structural characteristics that were found to be important for ALR inhibition can be seen in Figure 24. The aromatic ring for both the A and B-rings, unsaturation at Cα and Cβ, and hydroxyl groups at C2′ and C4′ of the A-ring are essential. Meanwhile, the introduction of a thioglycolic group at the A-ring will increase the ALR inhibition. Lastly, for PPAR-gamma activation, the methoxy group at C4 of the B-ring, as well as hydroxyl groups at C4′ and C5′ of the A-ring, will increase the activation (Figure 25).

**Figure 26:**
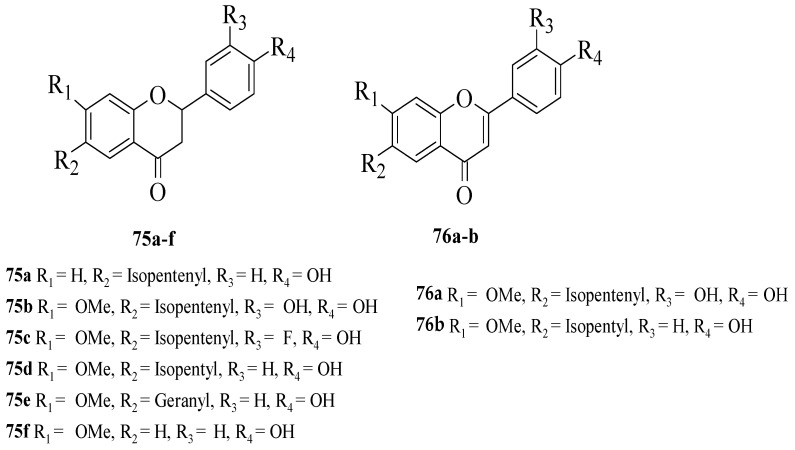
The chemical structures of studied flavonoids for PPAR-γ activation [62].

Du et al. synthesized several flavonoid derivatives to be tested as PPAR-γ agonists [62]. The synthesized flavonoids are shown in Figure 26. It was discovered that **75c**–**d** and **76b** (EC_50_ = 3.30, 13.61 and 3.55 μM, respectively) exerted higher activity compared to the control, bavachinin (EC_50_ = 18.74 μM). Then, the authors reported that removing the C7-methoxy group which can be seen in **75a** or removing the C6-isopentenyl chain and then replacing it with a geranyl chain (as can be seen in **75e**) can reduce the PPAR-γ activation. In contrast, the replacement of isopentenyl with isopentyl at C6 of the A-ring (**75d**) can improve the activity. The presence of an electron-donating group (**75b**) or electron-withdrawing group (**75c**) at C3′ was found to increase the PPAR-γ activation. Moreover, it was found that the activity was reduced by oxidising the C-ring of flavanone **75b** to flavone **75a**. Interestingly, oxidising the C-ring of flavanone **75d** to produce flavone **76b** boosted PPAR-γ activation.

**Figure 27:**
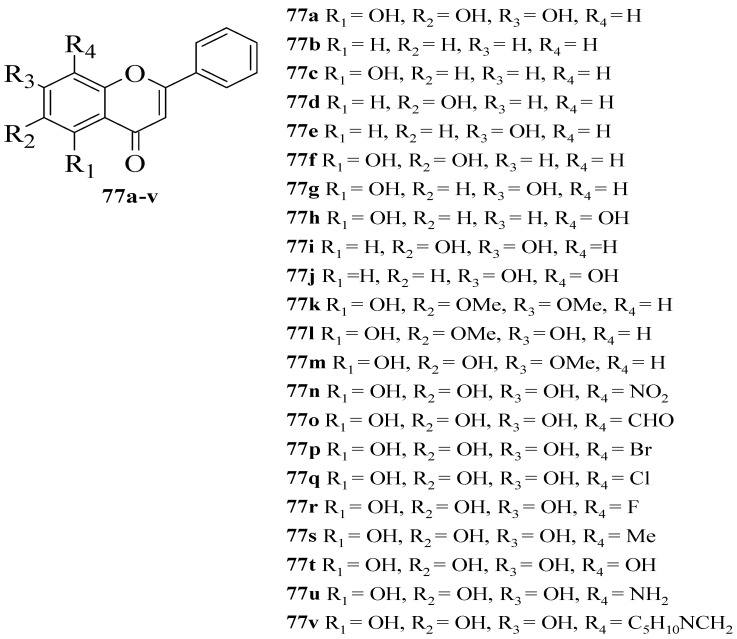
The chemical structures of synthesized flavone derivatives that were investigated for α-glucosidase inhibition [63].

Gao et al. synthesized several flavone derivatives and investigated the effect of the A-ring hydroxyl groups on α-glucosidase inhibition [63]. The flavones that were discussed are shown in Figure 27. It was reported that **77a** with trihydroxyl groups was the most potent inhibitor, with an IC_50_ value of 45 μM. Then, it was found that compounds **77b**–**j** in which there was an absence of any hydroxyl group at C5, C6, and C7 showed either weaker inhibition or inactivity. Most importantly, **77g** without C6-OH showed no inhibition, which led the authors to conclude that C6-OH is essential for inhibitory action. Next, **77t** was also shown to exert weaker inhibition when the hydroxyl group was added to position C8, despite having three hydroxyl groups pattern at C5, C6, and C7. Following that, the inclusion of an electron-withdrawing or electron-donating group at C8, as can be seen in **77n**–**q** and **77s**–**u**, resulted in either inactivity or became weak in terms of inhibition, despite the fact that **77r** lost a little amount of activity. Compound **77v** that contained bulky piperidino-methyl group at C8 was also found inactive. Hence, the researchers speculated that **77r**’s less bulky fluorine at C8 was responsible for its moderate activity compared to others. It can be concluded that C6-OH and substitution at C8 can influence the inhibition.

Sarian et al. isolated several flavonoids from the *Tetracera indica* (Houtt. ex Christm. and Panz.) Merr. (Dilleniaceae) and *Tetracera scandens* (Linn.) Merr. (Dilleniaceae) leaf extracts and then evaluated their antidiabetic effects via α-glucosidase and dipeptidyl peptidase IV (DPP-4) inhibition assays [64,65,66,67] (Table 1). The role of α-glucosidase is as discussed previously; meanwhile, the DPP-4 enzyme is involved in the breakdown of incretins such as glucagon-like peptide-1 (GLP-1), then inhibiting it and consequently lengthening the half-life of GLP-1, thereby extending the half-life of insulin. For α-glucosidase inhibition, the authors revealed that quercetin (**1**) possessing a catechol moiety showed the highest inhibition compared to other isolated compounds. Isoscutellarein (**78**) and kaempferol (**6**) with C4-OH showed weaker inhibition compared to **1**. Therefore, the catechol group, in which the hydroxyl groups at C3′ and C4′ of the B-ring, were thought to be crucial in α-glucosidase inhibition. Next, for DPP-4 inhibitory action, **1**, **78**, hypoletin (**79**), and **6** showed remarkable inhibition. The presence of hydroxyl groups can be considered to affect the inhibitory effect of DPP-4. The absence of a C2–C3 double bond and a 4-oxo group can further reduce the inhibition of α-glucosidase and DPP-4, according to the results of the study.

**Figure 28:**
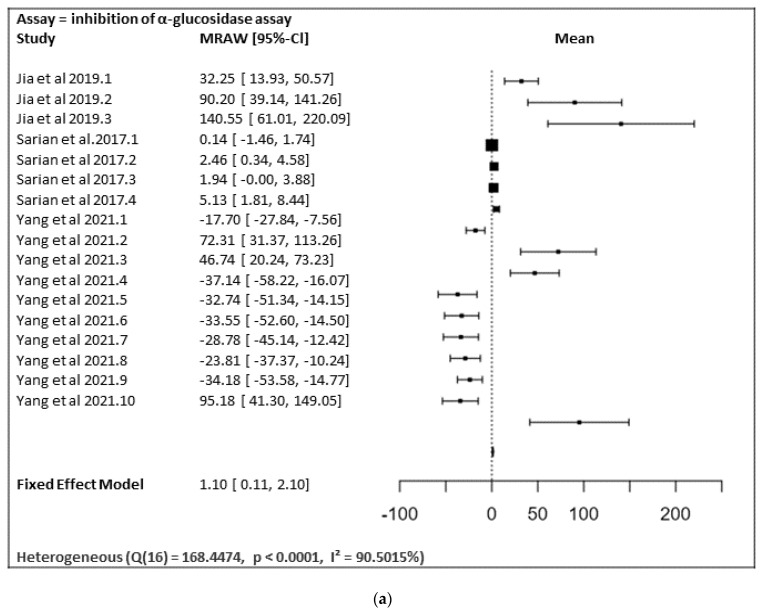

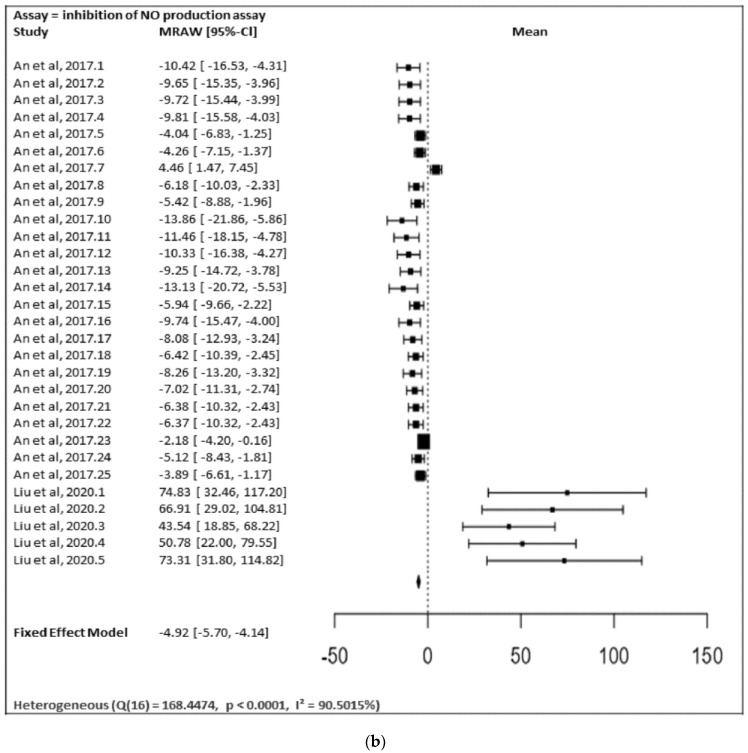
(**a**) Forest plot from meta-analysis of antidiabetic (inhibition of α-glucosidase) activity. (**b**) Forest plot from meta-analysis of anti-inflammatory (inhibition of NO production) activity.

In Figure 28a,b, the meta-analysis for anti-inflammatory and antidiabetic assays activities is shown based on the data given in Table 1. Due to incomplete data, only two studies from the inhibition of the NO production assay and three studies from the inhibition of the α-glucosidase of flavonoids were included in the meta-analysis, as summarized in Figure 28a,b, respectively.

Figure 28a shows the meta-analysis using a fixed effect model. It has revealed that the flavonoids, particularly those containing substitutions at positions 5, 3′, and 4′, showed a notable α-glucosidase inhibitory effect (MRAW = 1.1046 (95% CI: 0.1065–2.1027) overall. A total of k = 17 studies were included in the analysis. The observed standardized mean differences ranged from −37.1429 to 140.5504, with the majority of estimates being positive (59%). Therefore, the average outcome differed significantly from zero (z = 2.1690, *p* = 0.0301). According to the Q-test, the true outcomes appear to be heterogeneous (Q (16) = 168.4474, *p* < 0.0001, I² = 90.5015%).

Figure 28b shows the meta-analysis using a fixed effect model. It revealed that the flavonoids possessing substitutions at the 5, 3′, and 4′ positions of the A-ring and the B-ring, respectively, showed a notable inhibition of NO production activity (MRAW = −4.9200 (95% CI: −5.6975 to −4.1424). A total of k = 30 studies were included in the analysis. The observed standardized mean differences ranged from −13.8567 to 74.8310, with the majority of estimates being negative (80%). Therefore, the average outcome differed significantly from zero (z = −12.4014, *p* < 0.0001). According to the Q-test, the true outcomes appear to be heterogeneous (Q (29) = 155.3766, *p* < 0.0001, I^2^ = 81.3357%).

In contrast, the inhibition of the generation of leukotriene B4 (LTB4) by human neutrophils, nuclear factor kappa B (NF-κB) inhibition, the inhibition of the generation of leukotriene B4 (LTB4) by human neutrophils, pancreatic α-amylase inhibitory activity, rat lens aldose reductase (RLAR) inhibitory activity, human recombinant aldose reductase (HRAR) inhibitory activity, the advanced glycation end products (AGE) inhibitory activity assay, and the DPP-4 enzyme inhibitory assay were performed as only one study; therefore, a heterogenic analysis was not possible.

## 4. Analysis of the SAR of Flavonoids as Anti-Inflammatory and Antidiabetic Agents

As earlier described, inflammation is strongly associated with diabetic pathogenesis in both T1D and T2D. As a result, an antidiabetic drug possessing anti-inflammatory capabilities has the great potential to be a promising treatment for both T1D and T2D ailments. Flavonoids have been identified as one of several therapeutic therapies for diabetes mellitus due to their remarkable dual antidiabetic and anti-inflammatory capabilities. 

**Figure 29:**
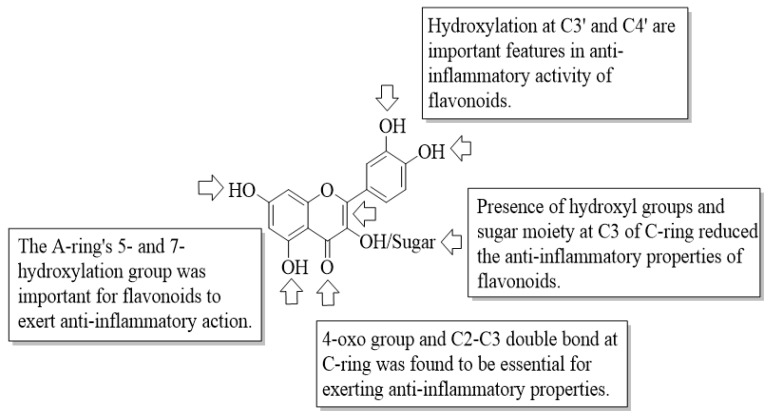
The SAR of flavonoids as anti-inflammatory agent.

According to the findings in this review (Table 2, Figure 29), hydroxyl groups at the C5 and C7 positions in the A-ring, as well as hydroxyl groups at the B-ring, especially C3′ and C4′, seem to be crucial for flavonoids to exert anti-inflammatory effects via a variety of mechanisms, including the inhibition of COX-2, LOX, LTB4, iNOS, NO, TNF-α, PGE_2_, and IL-8. Moreover, the C2–C3 double bond and C=O at C4 in the C-ring are also found to be important for the anti-inflammatory action of flavonoids. The inclusion of a sugar moiety and hydroxyl group, particularly at the C3 position of the flavonoids skeleton, on the other hand, can reduce anti-inflammatory activity. 

**Figure 30:**
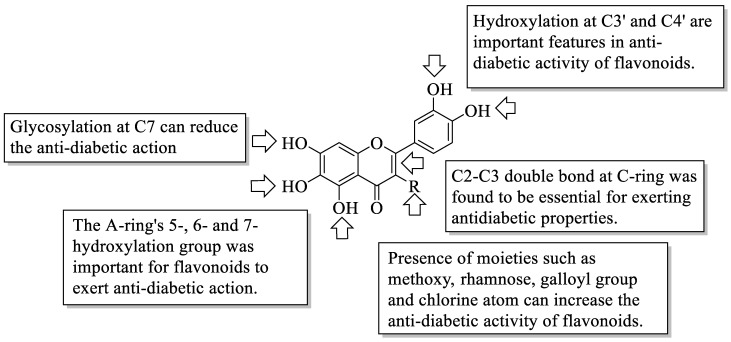
The SAR of flavonoids as an antidiabetic agent.

Similarly, according to Table 2 and Figure 30, numerous studies have been reported to demonstrate that hydroxyl groups at the C3′, C4′, C5, C6, and C7 positions are required for flavonoids to exhibit antidiabetic activity such as against glycogen phosphorylase, aldose reductase, AGE, and α-glucosidase. It has also been discovered that glycosyl and geranyl moieties instead of the hydroxyl group at C7 can reduce flavonoids’ antidiabetic effect. The presence of the C2–C3 double bond has also been observed to be critical for flavonoids’ antidiabetic activity. Furthermore, multiple investigations further reported that the substitution of hydroxyl groups at C3 with several functional group moieties such as methoxy, sugar-like rhamnose, galloyl group, and chlorine atom can increase the antidiabetic activity of flavonoids. 

**Figure 31:**
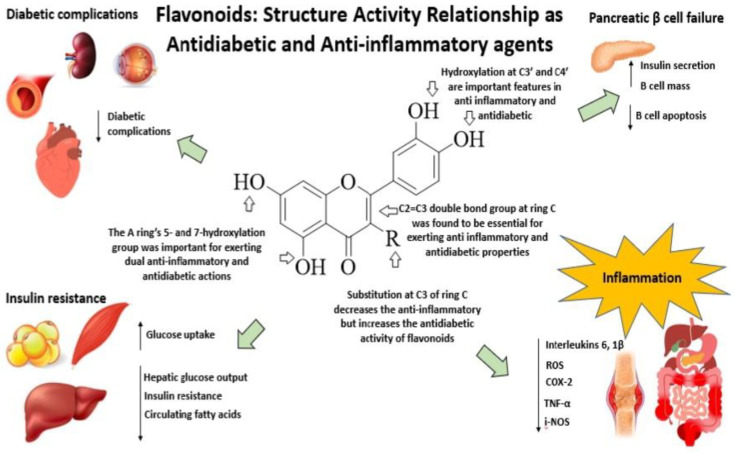
The SAR of flavonoids as dual action via anti-inflammatory and antidiabetic effects. Flavonoids have been reported to reduce diabetes complications, improve insulin secretion and pancreatic β cell mass, reduce β cell apoptosis, hepatic glucose output, insulin resistance, and circulating fatty acids, and downregulate ROS and inflammation markers such as IL-β and IL-6, Tnf-α, COX-2, and i-NOS.

Figure 31 illustrates the effect of flavonoids and their structure-activity relationship with antidiabetic and anti-inflammatory activities [9,11,14,15,16,17,18,19,20,21,22,23]. As a result, based on Figure 29 and Figure 30, flavonoids benefit from the presence of the C2–C3 double bond and the hydroxyl groups of the C3′, C4′, C5, and C7 positions due to their overlapping significant structural features for the manifestation of both anti-inflammatory and antidiabetic effects (Figure 31). However, it was discovered that substituting several moieties at C3 can lower the anti-inflammatory activity of flavonoids, whereas it can increase their antidiabetic activity (Table 2). Hence, the suitable bio-isosteres at the C3 position of the flavonoid scaffold possessing both antidiabetic and anti-inflammatory properties may help to discover potent therapeutic agents to treat both ailments efficaciously. 

## 5. Conclusions

Flavonoids are one of the major secondary metabolites of plants that have been reported to demonstrate various pharmacological effects including antidiabetic and anti-inflammatory activities. Since flavonoids comprise the same core scaffold, the functional variation is largely associated with the presence of different substituent groups in the different positions of the flavonoid’s skeleton. Flavonoid–protein (viz. enzymes, receptors, transporters, and transcription factors) interactions are important phenomena dictating the flavonoids’ beneficial distinct pharmacological properties. The relationship between the chemical constitution fragment and pharmacological effects has revealed that the presence of appropriate bio-isosteres as side chains can significantly affect the biological activity of flavonoids on the same target receptor. Hence, the structure–activity relationship analysis of flavonoids is important in understanding the dual mechanistic view of flavonoids in demonstrating the anti-inflammatory and antidiabetic effects. It can be concluded that for a flavonoid molecule to exert anti-inflammatory and antidiabetic activities together, the presence of a C2–C3 double bond (C-ring) and hydroxyl groups at positions C3′, C4′ (B-ring), C5, and C7 (A-ring) are essential structural requirements. Substitution at the C3 location, on the other hand, has been shown to lower flavonoids’ anti-inflammatory activity while increasing their antidiabetic activity. Therefore, the modification of these positions with the introduction of appropriate bio-isosteres will certainly play an important role in synthesizing more potent flavonoid-based drugs possessing essential pharmacophores to display equipotent antidiabetic and anti-inflammatory effects. Different in vitro assays using a variety of biological test systems should further be chalked out to confirm these flavonoids as safe antidiabetic and anti-inflammatory agents and help us understand various mechanisms that would equally alleviate hyperglycaemia and inflammation. Moreover, the knowledge of structure−activity relationships in these assays may further prove to be helpful in evaluating the potential of the in vivo antidiabetic and anti-inflammatory activities of flavonoids. A meta-analysis of the context of the antidiabetic and anti-inflammatory effects of flavonoids further established the role of these polyphenolic compounds as antidiabetic and anti-inflammatory agents. This review provides a theoretical foundation for the development of high-bioactive and low-toxicity flavonoid-based active pharmaceutical ingredients to tackle diabetes and inflammation concurrently.

## Figures and Tables

**Table 1:** In vitro anti-inflammatory and antidiabetic activities of flavonoids.

Anti-Inflammatory Activities								
References	Flavonoids		Assay	Negative Control	Activity of Flavonoids	Std Dev	Positive Control	Std Dev
[25]	Quercetin (**1**)		Suppressive action on the transcriptional activity of the COX-2 gene in human colon cancer DLD-1 cells Reporter gene assay	N/A	IC_50_ 10.5 μM	0.7	N/A	N/A
	Rhamnetin (**2**)				IC_50_ 18.6 μM	2.1		
	Genistein (**3**)				IC_50_ 20.7 μM	1.4		
	Eriodyctiol (**4**)				IC_50_ 22.0 μM	0.2		
	Luteolin (**5**)				IC_50_ 22.0 μM	0.4		
	Kaempferol (**6**)				IC_50_ 39.3 μM	2.1		
	Fisetin (**7**)				IC_50_ 47.9 μM	2.9		
	Phloretin (**8**)				IC_50_ 52.5 μM	3.4		
	Catechin (**9**)				IC_50_ 415.3 μM	25.4		
	Epicatechin (**10**)				IC_50_ 415.3 μM	17.0		
	Epigallocatechin (**11**)				IC_50_ >500 μM	-		
	Myricetin (**12**)				IC_50_ >500 μM	-		
[27]	**5**		Inhibition of the generation of leukotriene B4 (LTB4) by human neutrophils	N/A	IC_50_ 1.6 μM	0.3	Nordihydroguaiaretic acid (NDGA), IC_50_ 56.6 μM	2.5
	3′,4′-dihydroxy flavone (**14d**)				IC_50_ 1.7 μM	0.1		
	3′,4′,7-trihydroxy flavone (**16d**)				IC_50_ 2.0 μM	0.7		
	3′,4′,5-trihydroxy flavone (**15d**)				IC_50_ 2.9 μM	0.8		
	**1**				IC_50_ 4.0 μM	1.2		
[28]	**1**		Inhibition on rabbit reticulocyte 15-LOX-1	N/A	IC_50_ 4.0 μM	N/A	N/A	N/A
	**5**				IC_50_ 0.6 μM	N/A		
	Naringenin (**18**)				IC_50_ 250 μM	N/A		
	Hesperidin (**19**)				IC_50_ 90 μM	N/A		
	**10**				IC_50_ 60 μM	N/A		
	Taxifolin (**13**)				IC_50_ 25 μM	N/A		
	**1**		Inhibition on soybean LOX L-1	N/A	IC_50_ 4.5 μM	N/A	N/A	N/A
	**5**				IC_50_ 3.0 μM	N/A		
	**13**				IC_50_ 1000 μM	N/A		
[29]	**1**		Inhibitory effect on LTB4 production	N/A	IC_50_ 2.0 μM	N/A	N/A	N/A
	3′-O-methylquercetin (**20**)				IC_50_ 2.0 μM	N/A		
	Quercetin-3′-O-sulfate (**21**)				IC_50_ 2.0 μM	N/A		
[32]	**28**		Anti-inflammatory effect on murine macrophage cell line and gastric epithelial cell (GES-1)	N/A	IC_50_ 53.40 μM	N/A	N/A	N/A
	**29**				IC_50_ 120.98 μM			
	**30**				IC_50_ 10.73 μM			
[33]	Isoorientin (**26**)		Nuclear factor kappa B (NF-κB) inhibition	N/A	IC_50_ 8.9 μg/mL	N/A	Parthenolide, IC_50_ 0.9 μg/mL	N/A
	Orientin (**31**)				IC_50_ 12.0 μg/mL	N/A		
	Isovitexin (**32**)				IC_50_ 18.0 μg/mL	N/A		
	**26**		Inducible nitric oxide synthase (iNOS) inhibition		IC_50_ 48.0 μg/mL	N/A	Parthenolide, IC_50_ 0.18 μg/mL	N/A
	**31**				IC_50_ 54.0 μg/mL	N/A		
	**32**				IC_50_ 21.0 μg/mL	N/A		
[37]	Apigenin (**17c**)		Inhibition of NO production	7-Nitroindazole, IC_50_ > 100 μM	IC_50_ 23 μM	N/A	2-amino-5,6-dihydro-6-methyl-4H-1,3-thiazine Hydrochloride (AMT), IC_50_ 0.09 μM	N/A
	**5**				IC_50_ 27 μM	N/A		
	**18**				IC_50_ >100 μM	N/A		
	Apiin (**34**)				IC_50_ >100 μM	N/A		
	Galangin (**35**)				IC_50_ >100 μM	N/A		
	**1**				IC_50_ 107 μM	N/A		
[39]	**37**		Inhibition of NO production	N/A	IC_50_ 19.87 μM	0.21	Hydrocortisone, IC_50_ 3.83 μM	0.12
	**38**				IC_50_ 15.69 μM	0.16		
	**39**				IC_50_ 9.19 μM	0.07		
	**40**				IC_50_ 10.32 μM	0.08		
	**41**				IC_50_ 18.43 μM	0.19		
**Antidiabetic activities**								
References	Flavonoids	Assay		Negativecontrol	Activity of flavonoids	Std Dev	Positive control	Std Dev
[46]	Quercetagetin (3,3′,4′,5,6,7-Hexahydroxyflavone [44])	Glycogen phosphorylase inhibition		N/A	IC_50_ 9.7 μM	N/A	N/A	N/A
[47]	Chrysin (**17a**)	Rat lens aldose reductase (RLAR) inhibition		N/A	IC_50_ 8.5 μM	N/A	N/A	N/A
	Diosmetin 7-O-β-D-glucopyranoside (**45**)				IC_50_ 23.0 μM	N/A		
	Diosmetin (**17f**)				IC_50_ 8.5 μM	N/A		
	**17c**				IC_50_ 2.2 μM	N/A		
	**6**				IC_50_ 10.0 μM	N/A		
[50]	Sophoflavescenol (**46**)	Rat lens aldose reductase (RLAR) inhibition		N/A	IC_50_ 0.30 μM	0.06	Epalrestat, IC_50_ 0.07 μM	0.00
		Human recombinant aldose reductase (HRAR) inhibition		N/A	IC_50_ 0.17 μM	0.03	Epalrestat, IC_50_ 0.15 μM	0.01
		Advanced glycation end products (AGE) inhibitory activity		N/A	IC_50_ 17.89 μM	1.44	Aminoguanidine, IC_50_ 81.05 μM	0.35
[51]	**47**	α-glucosidase inhibition		N/A	IC_50_ 8.96 μM	0.90	Acarbose, IC_50_ 28.06 μM	0.82
	**48**				IC_50_ 82.47 μM	0.22		
	**1**				IC_50_ 75.80 μM	0.81		
	**49**				IC_50_ 1.05 μM	0.03		
	**50**				IC_50_ 3.76 μM	0.17		
	**51**				IC_50_ 2.57 μM	0.25		
	**52**				IC_50_ 3.02 μM	0.54		
	**53**				IC_50_ 2.99 μM	0.86		
	**54**				IC_50_ 3.22 μM	0.01		
	**55**				IC_50_ 155.57 μM	1.27		
[55]	**62**	Pancreatic α-amylase inhibition		N/A	IC_50_ 44 μM	3.0	Acarbose, IC_50_ 1.3 μM	0.2
[57]	**12**	α-Glucosidase inhibition		N/A	IC_50_ 11.63 μM	0.36	Acarbose, IC_50_ 0.59 μM	0.14
	Apigenin-7-O-glucoside (**63**)				IC_50_ 22.80 μM	0.24		
	**7**				IC_50_ 46.39 μM	0.34		
	Pinocembrin (**68**)	α-Glucosidase inhibition (Sucrase activity)		N/A	IC_50_ 0.39 μM	0.02	Acarbose	N/A
	Pinocembrin (**68**)	α-Glucosidase inhibition (Maltase activity)		N/A	IC_50_ 0.35 μM	0.021		
[62]	**75a**	PPAR-γ agonism		N/A	EC_50_ 47.07 μM	N/A	Bavachinin, EC_50_ 18.74 μM	N/A
	**75b**				EC_50_ 11.25 μM	N/A		
	**75c**				EC_50_ 3.30 μM	N/A		
	**75d**				EC_50_ 13.61 μM	N/A		
	**75e**				EC_50_ 114.33 μM	N/A		
	**75f**				Inactive	N/A		
	**76a**				EC_50_ 42.53 μM	N/A		
	**76b**				EC_50_ 3.55 μM	N/A		
[63]	**77a**	α-Glucosidase inhibition		N/A	IC_50_ 45 μM	N/A	N/A	N/A
	**77r**				IC_50_ 86 μM	N/A		
	**77t**				IC_50_ 960 μM	N/A		
	**77u**				IC_50_ 1000 μM	N/A		
[64]	**1**	α-Glucosidase inhibition			IC_50_ 4.92 μg/mL	7.06	Quercetin (commercial), IC_50_ 4.30 μg/mL	1.06
	Isoscutellarein (**78**)				IC_50_ 7.15 μg/mL	0.96		
	**6**				IC_50_ 12.19 μg/mL	4.63		
	Hypoletin (**79**)				IC_50_ 48.42 μg/mL	9.71		
	**1**	DPP-4 inhibition			IC_50_ 21.75 μg/mL	5.81	Sitagliptin, IC_50_ 24.51 μg/mL	1.01
	**78**				IC_50_ 22.32 μg/mL	1.52		
	**6**				IC_50_ 45.93 μg/mL	8.61		
	**79**				IC_50_ 34.89 μg/mL	7.44		

**Table 2:** Important structural characteristics of different classes of flavonoids as anti-inflammatory and antidiabetic activities. The up arrow means increasement and the down arrow means decreasement of the activity.

**Anti-Inflammatory Activity of Flavonoids**											
**References**	**A-Ring**				**B-Ring**				**C-Ring**		
	**C5**	**C6**	**C7**	**C8**	**C2′**	**C3′**	**C4′**	**C5′**	**C2**	**C3**	**C4**
[25]	OH ↑		OH ↑			OH ↑	OH ↑		C2=C3 ↑		C=O ↑
[26]						OH ↑	OH ↑		C2=C3 ↑	OH ↓	
[27]						OH ↑	OH ↑		C2=C3 ↑	OH ↓	
[28]	Catechol moiety ↑				Catechol moiety ↑				C2=C3 ↑		C=O ↑
[29]						Conjugation of OH ↓			C2=C3 ↑	Glucuronidation of OH ↓	
[30]	OH ↑	Glycosylation of OH or C ↑	OH ↑			OH ↑	OH ↑				
[31]				Glucopyranosyl ↑			OMe ↑		C2=C3 ↓	OH and sugar moiety ↓	
[32]	Methylation ↑ OH ↓										
[33]	Glycosylation ↑					OH ↑	OH ↑		C2=C3 ↑		
[34]	OH ↑				OH ↑	OH ↑	OH ↑	OH ↑	C2=C3 ↑	Sugar moiety ↓	
[35]						OH ↑	OH ↑		C2=C3 ↑		
[36]	OH ↑		OH ↑						C2=C3 ↑		C=O ↑
[37]	OH ↑		OH ↑	OMe ↑		OH ↑	OH ↑		C2=C3 ↑	OH ↓	
[38]						OH ↑	OH ↑				
[39]	OH ↑		OH ↑				OH ↑				
[40]	OH ↑		OH ↑						C2=C3 ↑	OH ↑	C=O ↑
[41]	OH ↑		OH ↑			OH ↑	OH ↑				
[42]					OH ↑ Conjugation of OH ↓	OH ↑ Conjugation of OH ↓	Substitution ↓				
**Antidiabetic activity of flavonoids**											
**References**	**A-Ring**				**B-Ring**				**C-Ring**		
	**C5**	**C6**	**C7**	**C8**	**C2′**	**C3′**	**C4′**	**C5′**	**C2**	**C3**	**C4**
[46]						OH ↑	OH ↑		C2=C3 ↑		
[47]			Sugar moiety ↓			OH ↑	OH ↑			OH ↓	
[48]	OH ↑		OH ↑ Glycosylation of OH ↓			OH ↑ Conjugation of OH ↓	OH ↑ Conjugation of OH ↓			Methylation of OH ↑	
[49]										OMe ↑	
[50]	OMe ↑			Prenyl group ↑		OH ↑	OH ↑				
[51]					Gallolyl moiety ↑ OH ↑	OH ↑	OH ↑	OH ↑			
[52]						OH ↑					
[53]			Rhamnosyl moiety ↑							Rhamnosyl moiety ↑	
[54]	OH ↑		OH ↑	OH ↑		OH ↑	OH ↑		C2=C3 ↑	OH ↑	
[55]	OH ↑		OH ↑			OH ↑	OH ↑		C2=C3 ↑	Cl ↑	
[56]			Bulky group and H bond acceptor ↑							Minor group and EDG ↑	
[57]			OH ↑								
[58]		OH ↑	Glycosylation of OH ↓			OH ↑		OH ↑	C2=C3 ↑	Galloylation of OH ↑ Glycosylation of OH ↓	
[59]	OH ↑	OH ↑	OH ↑				OMe ↑				
[62]		Isopentyl moiety ↑	Geranyl moiety ↓			EDG or EWG ↑					
[63]	OH ↑	OH ↑	OH ↑	OH ↓ F ↑							
[64]						OH ↑	OH ↑		C2=C3 ↑		C=O ↑
